# High-Resolution X‑ray Absorption Spectroscopy of Soluble Methane Monooxygenase: Room Temperature and Cryogenic Insights into Active Site Electronic Structure Modulation

**DOI:** 10.1021/jacs.6c13395

**Published:** 2026-10-15

**Authors:** Olivia McCubbin Stepanic, Sinjini Bhattacharjee, Christopher J. Pollock, Kara A. Zielinski, Rahul Banerjee, Derek B. Rice, Kushal Sengupta, John D. Lipscomb, Dimitrios A. Pantazis, Frank Neese, Lois Pollack, Serena DeBeer

**Affiliations:** † Max Planck Institute for Chemical Energy Conversion, Mülheim an der Ruhr 45470, Germany; ‡ Max-Planck-Institut für Kohlenforschung, Mülheim an der Ruhr 45470, Germany; § Cornell High Energy Synchrotron Source, Wilson Laboratory, Cornell University, Ithaca, New York 14853, United States; ∥ School of Applied Engineering and Physics, Cornell University, Ithaca, New York 14853, United States; ⊥ Department of Biochemistry, Molecular Biology, and Biophysics, University of Minnesota, Minneapolis, Minnesota 55455, United States; # Department of Chemistry and Biochemistry, Auburn University, Auburn, Alabama 36849, United States

## Abstract

Room temperature HERFD XAS (High Energy Resolution Fluorescence Detected X-ray Absorption Spectroscopy) data collected on the stable Fe­(III)­Fe­(III) (ox) and Fe­(II)­Fe­(II) (red) states of the soluble methane monooxygenase hydroxylase (sMMOH) from *Methylosinus trichosporium* (*Mt*) OB3b using laminar flow cells reveal structural differences with varied temperature, pH, and presence of regulatory protein MMOB. High quality room-temperature data on the X-ray radiation sensitive MMOH-ox:MMOB state shows two distinct radiative reduction events, confirming the presence of a so-called semimet (Fe­(II)­Fe­(III)) state in *Mt* sMMO, and for the first time presents the undamaged HERFD XAS spectra of MMOH-ox and MMOH-ox:MMOB. Clear differences in the whiteline and pre-edge of MMOH-red:MMOB appear between room temperature and cryogenic data, indicating the presence of cryogenic artifacts. In light of this new room temperature data, TD-DFT calculations based on optimized QM/MM models have been used to assign the structures of MMOH-red and ox states. MMOH-red and MMOH-red:MMOB have been assigned as 6,6-coordinate on the basis of pre-edge intensities, with minor perturbations in the ligand field seen upon MMOB binding. MMOH-ox:MMOB and MMOH-ox have been assigned as 6,6-coordinate with different protonation states at pH 7 on the basis of calculated models and changes in the experimental pre-edge energies and intensities for MMOH-ox:MMOB at pH 7.5. Together these results establish a revised structural and mechanistic framework for sMMO and demonstrate the potential of microfluidic HERFD XAS for capturing authentic enzymatic states.

## Graphical abstract

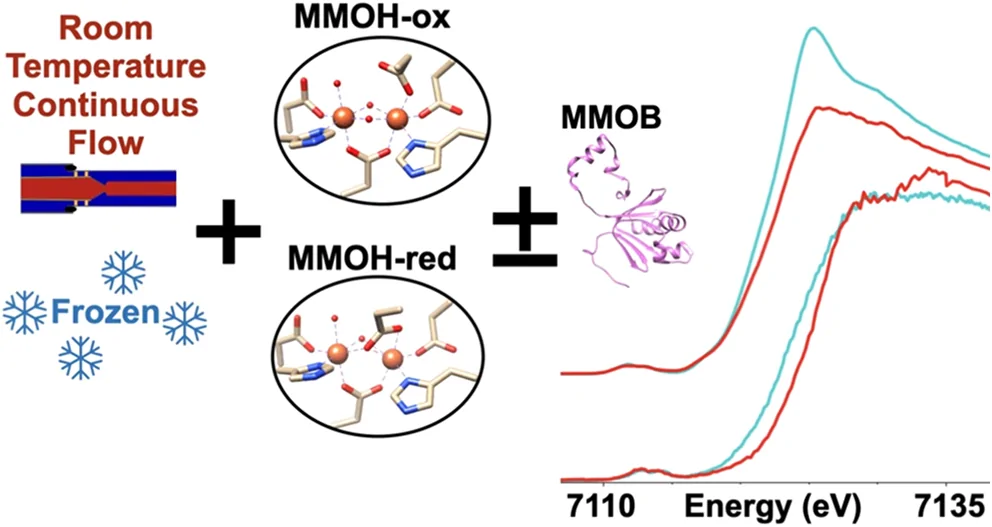

## Introduction

Soluble methane monooxygenase (sMMO) is a nonheme diiron enzyme that catalyzes the direct conversion of methane to methanol using molecular oxygen, a transformation that has attracted sustained mechanistic interest for over five decades.^1–4^ sMMO is generally understood to be minimally a 3 component protein system consisting of a hydroxylase (MMOH), a reductase (MMOR), and a regulatory protein (MMOB).^1–8^ MMOH possesses an α_2_β_2_γ_2_ architecture containing a diiron catalytic active site in each α subunit. MMOR is responsible for the 2-electron reduction of MMOH from its diferric resting state (MMOH-ox) to the diferrous reduced state (MMOH-red) from which the catalytic cycle starts.^9–12^ The reduced MMOH-red:MMOB complex then reacts with oxygen to form a series of intermediates including the diferric peroxo intermediate P and the high spin di-Fe­(IV) intermediate Q, which is responsible for direct H atom abstraction from methane.^13,14^ Subsequent oxygen rebound reaction generates methanol as the final product.^15^ MMOB has been observed via X-ray crystallography and CD/MCD spectroscopies to influence the active site in both the MMOH-red and -ox states.^16–19^ Further, MMOB is required for multiple turnovers, highlighting the importance of protein–protein interactions in controlling reactivity.^16,20–23^ However, the subtle geometric and electronic structural changes associated with MMOB binding remain incompletely understood, particularly under physiologically relevant conditions.

Structural and electronic characterization of sMMO is critical for understanding the catalytic mechanism of the enzyme. Among the available techniques, metal K-edge X-ray absorption spectroscopy (XAS) is particularly powerful because it provides element-specific information about oxidation state, coordination geometry, ligand field, and spin state.^24,25^ XAS studies under cryogenic conditions have previously been used to investigate the effect of MMOB and potential inhibitory protein MMOD binding at the diiron active site for the isolable states of sMMO from *Methylococcus capsulatus* (*Mc*) Bath.^8,26^ More recently, we have utilized HERFD (High Energy Resolution Fluorescence Detected) XAS to obtain higher resolution pre-edges for the *Methylosinus trichosporium (Mt)* Ob3B MMOH:MMOB complex in the MMOH-ox, MMOH-red and Q states under cryogenic conditions.^27^ These data have provided key evidence in support of an open core structure for Q. Although cryogenic measurements have greatly advanced the field, they also raise important questions regarding the extent to which frozen samples accurately reflect the structure and dynamics of the enzyme under native conditions.

The widespread use of cryogenic measurements across structural biology has been driven largely by their ability to mitigate radiation damage. In macromolecular crystallography, cryocooling revolutionized data collection by suppressing radical diffusion and secondary structural rearrangement induced by X-ray exposure.^28^ Consequently, the vast majority of protein crystal structures deposited in the PDB have been collected below 273 K. Only recently, with the emergence of X-ray free electron lasers (XFELs), has a renewed attention been directed toward measurements under near-physiological conditions.^29,30^ Comparative studies have since demonstrated that cryocooling can introduce artifacts in a variety of systems.^29,31–33^

Similar challenges exist in spectroscopic investigations of metalloenzymes. For nonheme iron enzymes in particular, detailed electronic structure characterization often relies on intrinsically low temperature techniques such as Mössbauer spectroscopy, electron paramagnetic resonance, and magnetic circular dichroism, all of which generally require frozen samples for these types of systems.^34,35^ X-ray spectroscopies are, in principle, compatible with both room temperature and cryogenic measurements; however, radiation damage remains a major limitation at ambient temperatures, often necessitating prohibitively large quantities of protein.^28^ As a result, most X-ray spectroscopic studies performed on metalloenzymes are done under cryogenic conditions.^24,36^

For dynamic multicomponent protein systems such as sMMO, the reliance on frozen samples is particularly concerning because temperature-dependent changes in protein structure and flexibility may directly influence catalytically relevant protein–protein interactions. Consequently, the geometric and electronic structures observed at cryogenic temperatures may not fully represent those present under physiological conditions. Addressing this challenge requires experimental approaches capable of minimizing radiation damage while maintaining the enzyme in solution at near-native temperatures.

Recent advances in microfluidic flow cell technology have enabled room temperature X-ray crystallographic and scattering studies on a variety of biological systems by rapidly refreshing the sample volume exposed to the beam using a flow cell.^37–40^ The main benefit to this approach is the reduction of the absorbed dose per unit volume. Although this approach has become increasingly common, it has not been widely adopted for X-ray spectroscopy. Given the high radiation sensitivity of metalloenzymes, this combination is particularly promising for X-ray spectroscopic studies. Currently, radiation damage is managed by attenuating the X-ray beam to lower the dose rate and by cryogenically cooling the samples to raise the total acceptable dose. The low dose rate, however, comes at the cost of extremely long data collection times. With the continuous flow approach in a flow cell, refreshing the exposed volume keeps the per-volume dose low even at higher incident flux, which can substantially shorten data collection times.

Preliminary room temperature X-ray spectroscopic studies from our group and others have demonstrated the promise of these approaches.^41–43^ Compared with conventional flow cell approaches, the flow cells utilized in this study reduce sample consumption by approximately an order of magnitude, making HERFD XAS increasingly practical for precious biological samples, while also reducing the absorbed dose per unit volume. This decrease in absorbed dose via exposure time also has the potential to mitigate the need to reduce the X-ray flux (ph/s) as is typically required for cryogenic, radiation sensitive samples, making room temperature data collection faster than traditional cryogenic methods.

Herein, we present a comparative Fe K-edge HERFD XAS study of sMMOH in both MMOH-ox and MMOH-red states under cryogenic and room temperature conditions, revealing substantial freezing-induced geometric/electronic changes at the diiron active site. To our knowledge, all previous XAS studies on sMMO were performed under cryogenic conditions, making this the first Fe K-edge HERFD XAS data on sMMOH in the MMOH-ox and MMOH-red states under room temperature, catalytically relevant conditions. By employing flow to mitigate radiolytic damage, we demonstrate that the majority of previous cryogenic measurements of MMOH-ox were compromised by radiation damage, and we report the first undamaged Fe K-edge HERFD XAS spectrum of the MMOH-ox:MMOB complex. Correlation of the room-temperature solution data with TD-DFT/QM/MM calculations enables structural assignments, including the influence of the regulatory protein MMOB. Together, these results provide a framework for reassessing the existing spectroscopic literature on sMMO and highlight the broader challenges associated with interpreting frozen state measurements of metalloenzymes. By integrating XAS with laminar flow sample delivery, including passage through a microfluidic mixing element enabling future time resolved applications, we capture electronic snapshots of sMMO under biologically relevant conditions without requiring extended data collection times. Given the extensive effort devoted to elucidating the sMMO catalytic pathway, direct comparison above and below the freezing point offers a route to resolve longstanding controversies surrounding the electronic and geometric structures of catalytic intermediates.^16,17,20,44–47^

## Experimental Section

### Sample Preparation

*Methylosinus trichosporium* *(Mt)* OB3b MMOB, MMOH and MMOR were purified according to previously described protocols.^27,48^ All samples consisted of 500 μM MMOH, 1 mM MMOB, and 40 μM MMOR in a 100 mM MOPS buffer at a pH of 7.0 unless otherwise stated. Glycerol (3% v/v) was present in solution as a cryoprotectant to protect the proteins from freeze/thaw cycles during transport. A stock solution of 367 mM NADH in 20 mM Tris buffer (pH 8.5) was used to reduce MMOH to the red state. Cryogenic samples of MMOH-red and MMOH-ox were prepared as previously described, using a prechilled sample cell followed by direct freezing with liquid nitrogen.^49^

### Flow and Sample Delivery

As the room temperature HERFD data were collected over multiple beamtimes, there are minor differences in the flow cells used for data collection. The schematics of both flow cells are shown in the SI (Figure S1). Both utilize the same thin-walled quartz glass (800 μm ID, 840 μm OD, Hilgenberg GmbH) for the observation region. One flow cell is made for coflowing the sample and the sheath, such that the sheath fully surrounds the sample stream to prevent sample cell fouling. It is fabricated with a single polished capillary (100 μm ID, 360 μm OD, Molex LLC) that is centered within the quartz with custom Kapton centering spacers.^37^ The other flow cell is a microfluidic mixing cell, incorporating a cone-opening Kenics device that can rapidly combine two species for reaction initiation.^40,41^ After passage through the microfluidic mixer, the mixed stream was sheathed by (anaerobic) buffer and flowed into a larger diameter capillary for measurement. For this work, the Kenics was only used to combine sample (protein) with buffer, resulting in the sample concentration being halved. Flow rates were adjusted to ensure the sample stream width, sample flow speed, and X-ray residence time were the same between both devices.

The sheath flow was delivered with an Elveflow OB1 Flow Controller (Elvesys) pressurized with helium gas. The He continuously filled the headspace of the sheath reservoir tube, maintaining the anaerobicity of the MOPS buffer (pH 7). The sample flow was delivered with HPLC pump(s) (LC-20AD and LC-30AD, Shimadzu Corporation). For the coflow device, a single HPLC pump was used. For the Kenics flow cell, two HPLC pumps were used, one for each port. For both devices, the protein sample was loaded into a stainless-steel reservoir (APT Technologies) with a Teflon plug, and the HPLC flow pushed against the Teflon plug for sample delivery. The reservoir was rinsed with a solution of dithionite and then deoxygenated buffer prior to loading the protein sample for MMOH-red samples. For the Kenics flow cell, an injection valve (Valco Instruments Co. Inc.) was used to prevent the introduction of bubbles to the flow cell. For the second Kenics line, MOPS buffer (pH 7) was directly flowed through the HPLC unit and delivered to the flow cell. Elveflow Bronkhorst flow meters were used to monitor flow rates. For the anaerobic measurements, all components on both the sample line and mixing buffer line were purged with nitrogen and subsequently placed inside an argon-filled glovebag to ensure an anaerobic environment. The fluidics schematics for the ambient and anaerobic measurements are depicted in Figures S2 and S3, respectively. Flow conditions are described in Table S1.

### High-Energy Resolution Fluorescence Detected X-ray Absorption Spectroscopy

HERFD XAS data on in situ samples were collected at the PIPOXS beamline at CHESS (Cornell High Energy Synchrotron Source) operating at 125 mA and 6 GeV. A UV/vis spectrometer was installed for the simultaneous collection of HERFD XAS and UV/vis spectra. The UV/vis data were utilized for monitoring flow stability, as described previously (Figure S4).^41^ The incident beam was adjusted to 400 × 400 μm to match the diameter of the sample stream and the UV/vis beam, such that the two spectroscopies probe the same sample volume, and that the entirety of the sample stream is probed by the X-ray beam. The incident energy was selected using a cryogenically cooled Si(311) double crystal monochromator with a flux of 5.2 × 10^11^ ph/s, and the internal energy calibration was performed by assigning the first inflection point of an iron foil to 7111.2 eV. The fluorescence from the sample was detected utilizing five spherically bent Ge(440) crystals aligned on intersecting Rowland circles, in combination with a Pilatus 100K detector. The spectrometer energy was set to the maximum of the Fe Kα_1_ emission line. Data were collected at 18 °C (291 K).

Cryogenic samples were measured at the PIPOXS beamline at CHESS and at the ID26 Beamline at ESRF (European Synchrotron Radiation Facility). Cryogenic experiments at PIPOXS were performed under the same conditions as the in situ experiments reported above, with a liquid nitrogen flow cryostat and frozen samples rather than the flow apparatus. A cryocooled Si(311) double crystal monochromator was used for all data measured at 77 K to maintain high resolution of pre-edge data.

Additional damage study experiments at ID26 occurred with the ring operating at a current of 196 mA (7/8 multibunch) and an energy of 6 GeV. The incident beam spot was 100 × 200 μm. A Si(111) double crystal monochromator was used to select incident energy, and the internal energy calibration was performed by assigning the first inflection point of an iron foil to 7111.2 eV. Incident flux was 1 × 10^13^ ph/s in absence of attenuating aluminum foils. The flux after attenuators was determined using the percent transmission at 7.15 keV given the sum of foil thicknesses, and was converted to dose on the basis of the 100 × 200 μm spot size, a penetration depth of 0.4 mm and a conversion factor of 1 7.15 keV photon = 1.15 × 10^–15^ Joules. The fluorescence from the sample was detected utilizing four spherically bent Ge(620) crystals aligned on intersecting Rowland circles, in combination with a Pilatus 100K detector. The spectrometer energy was set to the maximum of the Fe Kβ_1,3_ emission line. All ESRF data were collected at 30 K utilizing a liquid helium flow cryostat.

Damage studies on in situ samples were performed by flowing at different rates to achieve different dose (J/kg) at the sample. Data collected on sMMO in situ at doses above 10^4^ J/kg utilized a Si(111) monochromator to maximize flux (4.05x 10^12^ ph/s) and modulated flow rates to increase exposure time and consequently fluence. Residence times of 0.25 to 3 s were collected, resulting in a fluence range of 6.4 × 10^12^ to 7.5 × 10^13^ ph/mm^2^. Incident flux (ph/s) was determined using an ion chamber in the X-ray beam path directly before the sample, and was converted to dose on the basis of residence time, the 400 × 400 μm^2^ spot size, a penetration depth of 0.4 mm, and a conversion factor of 1 7.15 keV photon = 1.15 × 10^–15^ Joules.

Damage studies on cryogenic samples were performed by collecting successive edge scans (7105 to 7135 eV) to determine the dwell time per spot. In some cases (in particular for MMOH-ox samples), scans were collected on multiple spots and averaged to improve signal to noise ratio. Further information is available in the SI (Figures S5 and S6). Incident flux and dose were determined as above, where dwell time was used as residence time.

All spectra were collected in 0.25 eV steps from 7105 to 7140 eV and are shown with a Savitzky-Golay filter using a second order polynomial and 7 point (1.75 eV) smoothing (unsmoothed spectra shown in Figure S7). Spectra were normalized using scans of the same sample from 7100 to 7400 eV with 5 eV steps, except in the case of frozen MMOH-ox and MMOH-ox:MMOB samples, for which the room temperature long scans of each respective sample were used. Reproducibility of samples was confirmed by collecting data at multiple separate beamtimes. Spectra pre-edge regions were fit using pseudo-Voigt peaks, as shown in Figure S8.

### QM/MM Setup and Calculations

The room temperature XFEL crystal structure of *Mt* OB3b MMOH-ox:MMOB (6YD0.pdb, 1.95 Å resolution) was used as the starting point for QM/MM models of the sMMOH:MMOB complex.^16^ A cryocooled crystal of *Mt* OB3b MMOH-ox alone reported by Jones et al. (6VK6.pdb MMOH-ox,1.52 Å resolution) was used as the basis for QM/MM models of sMMOH alone, as no high resolution structures of *Mt* OB3b sMMOH at room temperature are available.^23^ The two protein monomers were explicitly solvated using a 15 Å water box and neutralized with an appropriate number of counterions (see Figure S9). The protonation states of the active site ligands (His and Glu) were manually assigned. All the oxygens coordinating to irons (the terminal and the bridging oxygens) were initially modeled as waters. The metal centers along with the protein and water ligands were explicitly defined using QM derived MK-RESP charges and custom force field parameters (see SI).^50–52^ The rest of the system was assigned standard Amber partial charges and force fields for the protein (Amberff14SB),^53,54^ waters (TIP3P)^55^ and ions (Joung-Cheatham) respectively, using AmberTools.^56,57^ The MM setups were then systematically minimized and equilibrated using the Amber20 program package.^56^ The final equilibrated MD snapshot(s) were used for generating the initial structures for all QM/MM calculations. In order to preserve the structural integrity of the diiron core close to the crystallographic geometry (Figures S11 and S12) and to avoid unphysical force-field distortions of the protein matrix, weak positional restraints were applied to the active site residues during the MD equilibration. The final equilibrated snapshot was used for each of the MMOH-red, MMOH-ox, MMOH-red:MMOB and MMOH-ox:MMOB QM/MM models thereby isolating the electrostatic effects of MMOB binding only. We acknowledge that a single snapshot does not fully capture the conformational heterogeneity of the protein environment which may be more crucial for mechanistic studies. However, given the predominantly local nature of spectroscopic signatures computed here, we consider this snapshot a physically justified reference for comparing the different QM/MM models. Further details on the QM/MM setup are provided in the SI (Figures S9–S15 and Table S2).

The QM/MM computations were performed using the multiscale module of ORCA 6.0, employing the electrostatic embedding approach.^58,59^ A full discussion of computational methodology is available in the SI. In brief, for all the QM/MM models, the QM regions were defined based on the first sphere residues along with those residue segments which interact with the first coordination sphere through H-bonds. The final QM regions consisted of ca. 250 atoms (Figure S13). For MMOH-ox, the protonation states of the solvent derived ligands in the active site are defined based on previous work by Schulz et al.^47^ All MMOH-ox models are 6-coordinate at both irons (“6,6”), with a terminal water and varying protonation states of the two bridging solvent derived oxygens: (H_2_O)-Fe-(μOH)_2_-Fe (models Ox1 and Ox1B) and (H_2_O)-Fe-μ­(OH)­μ­(O)-Fe (models Ox2 and Ox2B). The models for MMOH-red and MMOH-red:MMOB (Red1­(B), Red2­(B), and Red3­(B)) were constructed by incorporating the glutamate shift and varying the number and position of coordinating waters. This yielded 6,6; 5,6 and 5,5 coordination spheres for the iron centers, respectively. All QM/MM geometry optimizations were carried out without constraints using the B3LYP functional,^60^ as used in prior publications to provide trends in transition metal pre-edge features,^47,61^ and def2-TZVP basis set^62^ including the resolution of the identity (RI) approximation and D3BJ dispersion corrections.^63,64^ A table of the calculated spin populations of all models is provided in the SI (Table S2). X-ray absorption spectra were calculated with the TDDFT method employing the Tamm-Dancoff approximation, the B3LYP functional and def2-SVP for C and H atoms def2-TZVP for N, O and Fe atoms in the QM region. The RI and chain of spheres (RIJCOSX) approximation along with corresponding auxiliary basis set (def2/J) were employed to speed up the calculations.^65,66^ The XAS spectra of each Fe center were calculated with 100 roots, and the spectra of each model were plotted by adding the individual spectra of the two Fe ions and using a peak Gaussian broadening of 1.0 eV using the *orca_mapspc* utility. In order to compare with the energies and normalized intensities of the experimental spectra, all computed intensities were divided by 18 and a precomputed energy shift of 150.85 eV was applied to the calculated spectra, as previously determined by the difference between calculated XAS spectra and experimental HERFD XAS pre-edge energies for molecular iron complexes.^27,47^

In order to evaluate the local iron geometries, a continuous shape measurement analysis was performed using the SHAPE V2.1 software.^67^

## Results

### HERFD XAS of MMOH-Red States

Fe K-edge HERFD XAS of MMOH-red at pH 7 both in the presence and absence of MMOB, collected at 77 and 291 K, are shown in Figure 1A-B. The spectra consist of two regions of interest: the intense rising edge (7120–7130 eV), which results primarily from dipole allowed 1s to 4p transitions, and the significantly less intense pre-edge region (7110–7116 eV, Table 1), resulting from formally dipole forbidden 1s to 3d transitions that gain intensity due to 3d-4p mixing in noncentrosymmetric coordination environments.^24,25^ The rising edge of all measured species of MMOH-red share a first inflection point of 7123.3 eV. The most dramatic spectral differences between species of MMOH-red are seen at the so-called whiteline, or rising edge maximum, at ∼7125 eV, in which the MMOH-red:MMOB at 291 K is a clear outlier and lacks the sharp feature seen in the other three spectra.

**Table 1:** Experimental and Calculated Fit Parameters for HERFD XAS Pre-Edges of MMOH-Red and MMOH-ox with and without MMOB

		experimental		model 1^a^		model 2^a^		model 3^a^	
sample	temperature (K)	IWAE (eV)	area (a.u.)	IWAE (eV)	area (a.u.)	IWAE (eV)	area (a.u.)	IWAE (eV)	area (a.u.)
MMOH-red	291 K	7112.01	10.9	7112.19	14.4	7112.19	17.6	7112.23	22.7
	77 K	7112.09	13.6						
MMOH-red:MMOB	291 K	7112.20	12.7	7112.20	13.5	7112.19	19.3	7112.21	17.7
	77 K	7112.00	13.3						
MMOH-ox	291 K	7113.35	15.9	7113.07	11.9	7113.60	17.0		
	77 K	7113.38	14.7						
MMOH-ox:MMOB	291 K	7113.18	15.8	7113.18	13.1	7113.53	17.3		
	77 K	7112.97	14.0						
MMOH-ox:MM0B pH 7.5	291 K	7113.33	16.7						

a For individual model names used throughout the text, please refer to Figure 3. For this table, models utilize the same number as Figure 3 and follow the same oxidation state and presence/absence of MMOB as written in the sample column.

**Figure 1:**
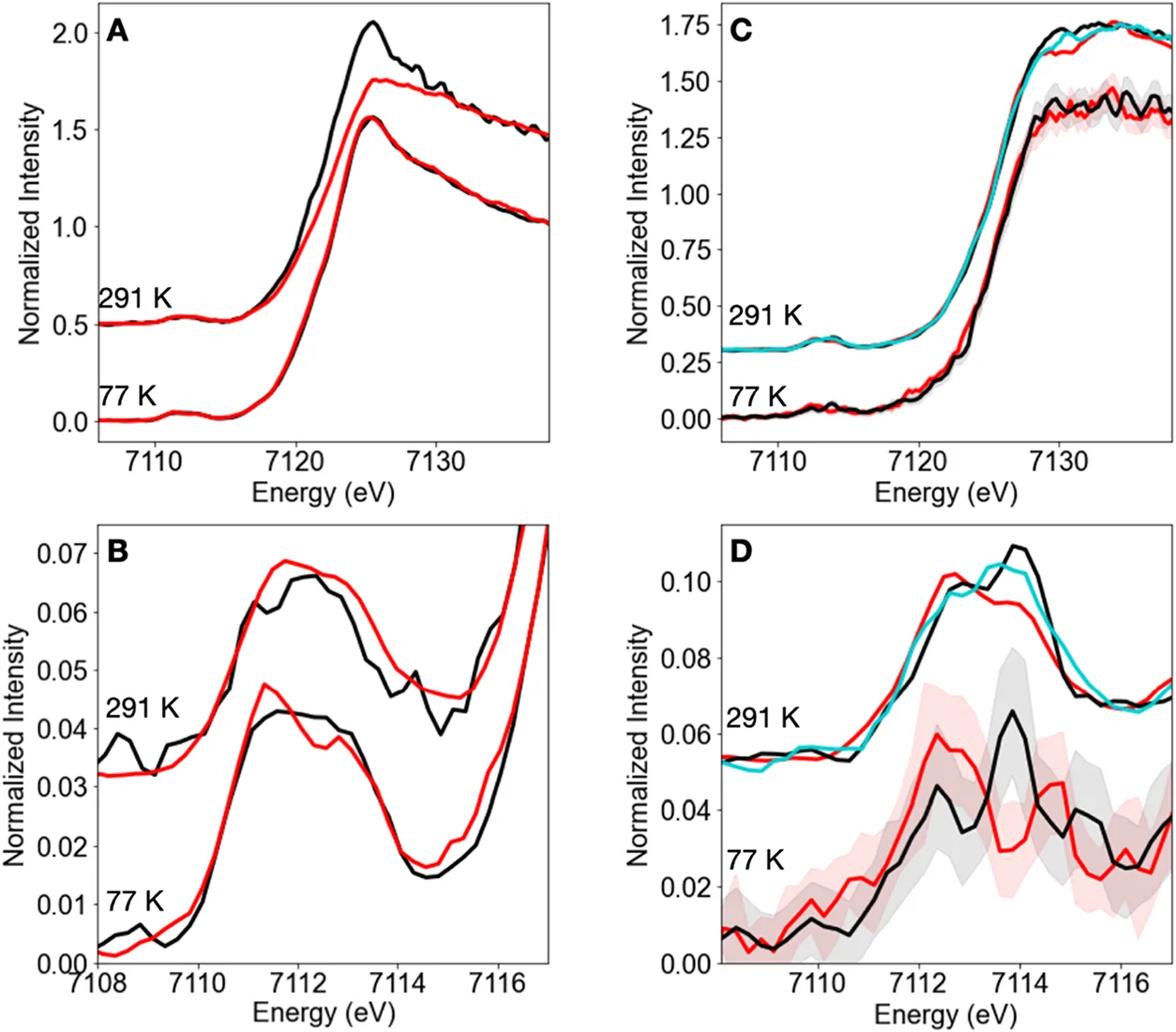
(A) HERFD XAS at pH 7.0 of MMOH-red alone (black) and with MMOB (red) collected at 291 and 77 K with zoomed pre-edge region below (B). (C) HERFD XAS spectra of MMOH-ox alone (pH 7, black) and with MMOB (pH 7, red; pH 7.5 light blue) collected at 291 K and at 77 K, with zoomed-in pre-edge region below (D). Error bars are shown for 77 K MMOH-ox alone and with MMOB in the respective colors of the spectra. 77 K data for MMOH-ox alone and with MMOB are not analyzed quantitatively due to the low signal-to-noise ratio of these data. All spectra are shown with 1.75 eV smoothing.

In order to quantitatively discuss the changes to the pre-edge upon MMOB binding and sample freezing, the pre-edge regions were fit using pseudo-Voigt peaks. The results of these fits are summarized in Table 1 (corresponding fits and uncertainties, Figure S8 and Table S3). For MMOH-red at 291 K, the pre-edge has an intensity weighted average energy (IWAE) of 7112.0 eV. For MMOH-red:MMOB at 291 K, the IWAE is shifted to 7112.2 eV, consistent with a broadening of the pre-edge feature. Clear changes also occur in the pre-edge region upon freezing for both MMOH-red alone and MMOH-red:MMOB, the most visible of which is the −0.2 eV shift in the IWAE of the MMOH-red:MMOB pre-edge upon freezing. For MMOH-red alone, an increase in the pre-edge area from 10.9 to 13.6 is seen upon freezing, while the IWAE remains the same within error (Figure S16). These changes indicate that MMOB perturbs the local ligand field at the active site at room temperature, while any apparent cryogenic changes upon MMOB binding are within experimental precision. Further, clear electronic structure modulations exist upon cryocooling of MMOH-red:MMOB relative to the 291 K electronic structure.

### HERFD XAS of MMOH-ox States

Fe K-edge HERFD XAS of MMOH-ox at pH 7 both in the presence and absence of MMOB collected at 77 and 291 K are shown in Figure 1C–D. The K-edges of all MMOH-ox spectra presented here are shifted by ∼3.5 eV to higher energy compared to the MMOH-red spectra, consistent with an increase in oxidation state. We note, however, that the ∼3.5 eV shift is larger than the ∼2 eV shift often reported for a one-unit change in Fe oxidation state in the absence of significant structural changes,^68,69^ and may therefore reflect additional contributions from changes in local geometry, protonation state, and/or magnetic coupling between the iron centers, as discussed further in the computational section. In addition, in the present data, both at 291 and 77 K, we observe that the rising edge inflection at ∼7125 eV is ∼1.3 eV higher in energy than the previously reported values for MMOH-ox:MMOB.^8,27^ Extensive damage studies on MMOH-ox:MMOB, detailed in the SI (Figures S5–S7) and summarized in Figure 2, revealed two distinct damage events occurring at doses differing by 2–3 orders of magnitude. These observations suggest that all previously reported data likely corresponds to the Fe­(II)­Fe­(III) semimet form of the enzyme.^8,26,27,44^ Herein, we have shown that the Fe­(III)­Fe­(III) species can only tolerate a dose of ∼1 × 10^4^ J/kg. The 77 K MMOH-ox:MMOB data, which is the result of five 10 s scans per spot on ∼100 spots, with an incident flux of 2.4 × 10^11^ ph/s (6% of the full 4 × 10^12^ ph/s beam), has 9.7× the noise of the 291 K data in the pre-edge region. An individual 10 s scan has an average of 1.4 counts (38 cts/s) above the edge. Given that 10 s of exposure has a total scan time of ∼25 s with motor movements, and that the incident beam could be attenuated to 30% rather than 6% to collect a single 10 s scan per spot, we estimate 65 h would be required to collect a spectrum of quality comparable to the data collected at 291 K. In contrast, by utilizing the flow cell setup the flow speed could be varied to decrease the effective dose while maintaining the full incident X-ray beam, resulting in roughly 200 cts/s above the edge and a data collection time of ∼3 h per spectrum.

**Figure 2:**
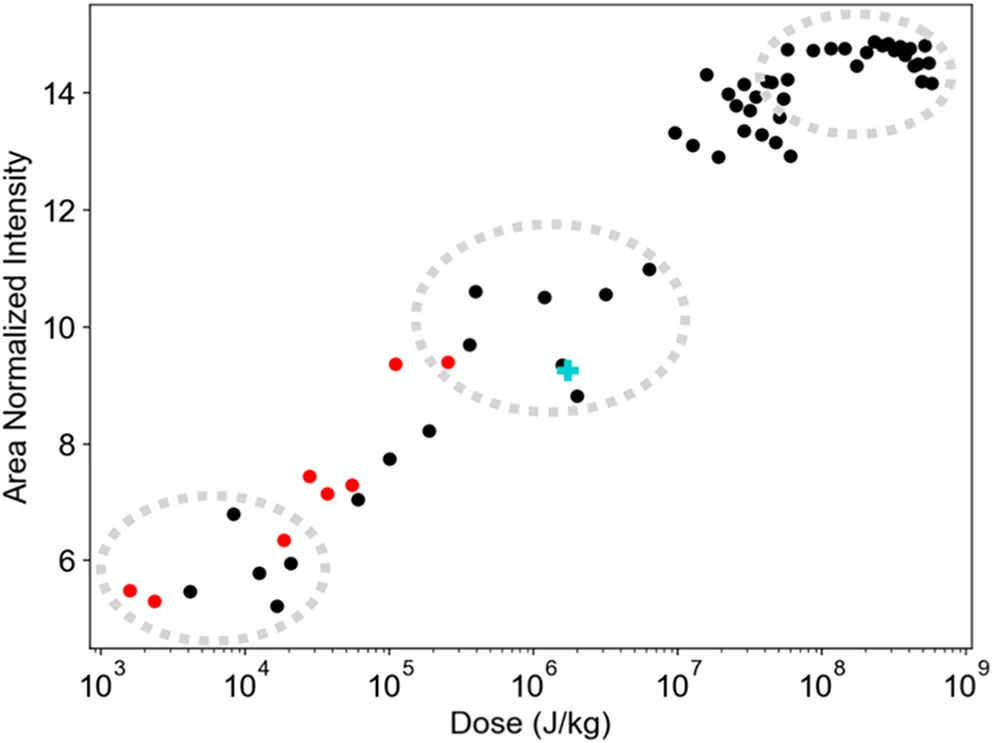
Measurement of normalized intensity at 7123 eV as a function of dose to monitor damage events. As the edge shifts to lower energy, the intensity at 7123 eV increases. Data points collected at 291 K are shown in red, while data collected at cryogenic temperatures are shown in black. The published data from Castillo 2017 is shown as a light blue plus (reproduced from^27^, copyright 2017 American Chemical Society). Gray dashed circles highlight the approximate plateau regions (see SI for details).

As shown in Figure 1C–D, the MMOH-ox and MMOH-ox:MMOB spectra are all similar above 7116 eV (at both 291 and 77 K), with slight modulations in the rising edge energy and whiteline shapes observed. In contrast, much more pronounced differences are observed in the pre-edge region. At both 291 and 77 K, the binding of MMOB to MMOH-ox modulates the local ligand field at the iron site, resulting in a decrease of the IWAE and an inversion in the pre-edge intensity pattern, seen in the areas of the ∼7112.7 and ∼7114.0 eV pre-edge features, summarized in Table S3. The low S/N ratio of the 77 K pre-edges precludes analysis of cryotemperature-dependent changes. MMOHox:MMOB at a pH of 7.5 (291 K) was also measured in order to allow for more direct comparison of these HERFD XAS data to the XFEL crystallographic data presented by Srinivas et al.^16^ The pre-edge spectrum of pH 7.5 MMOH-ox:MMOB cannot be replicated from a mixture of the spectra of pH 7 MMOH-ox:MMOB and MMOH-ox alone, and the pre-edge shape of pH 7.5 MMOH-ox:MMOB is sufficiently distinct from pH 7 MMOH-ox alone to rule out the pH 7.5 spectrum being a result of loss of MMOB binding (Figures S16 and S17). Further, the dissociation constants of MMOH-ox:MMOB complex show only a marginal increase with increasing pH, and the complex is expected to be fully intact given the protein concentrations used in this experiment.^70^ However, the IWAE for MMOH-ox:MMOB (7113.3 eV) at pH 7.5 is more similar to MMOH-ox alone at pH 7.0 than its MMOB-bound counterpart. A shift of the pre-edge IWAE to higher energy upon removal of MMOB or increase in pH may point to modulation of the protonation state of the diiron cluster between the MMOB bound and unbound states, a theory that can be further explored using computational models (*vide infra*).

### QM/MM Calculations on Select Models

In order to further investigate the active site structures of MMOH-red and MMOH-ox both with and without MMOB under ambient conditions, TD-DFT calculations were performed using QM/MM models starting from the 293 K XFEL structure of MMOH-ox:MMOB (6YD0, 1.95 Å, *Mt* OB3b), and from the 100 K structure of MMOH-ox alone (6VK6, 1.52 Å, *Mt* OB3b). Due to potential artifacts induced by freezing, and the fact that we were unable to obtain high-quality damage-free data on frozen MMOH-ox, we have primarily utilized the room temperature data for comparison.

Both MMOH-red alone and MMOH-red:MMOB have previously been assigned as 5-coordinate and roughly square pyramidal at both irons in the active site because of the relatively low ligand field transition energies seen in MCD (4.2 K) of frozen samples,^17–19^ although crystal structures reliably show a 6,6 coordination sphere,^16,23^ and a prior partial fluorescence yield (PFY) XAS measurement suggested a 5,6 coordination sphere on the basis of the measured pre-edge area compared to model complexes.^20^ The intensity of the pre-edge depends largely on p-d mixing and thus deviation of the local geometry from centrosymmetry, and as such both a decrease in coordination number and distortion from rigorously octahedral geometries can result in increased pre-edge intensity.^71,72^ Here, all three types of coordination spheres (Figure 3, and Table 1) were examined to investigate the active site structure of MMOH-red, both in the presence and absence of MMOB. A detailed description of the QM/MM models and optimized geometries is provided in the SI.

**Figure 3:**
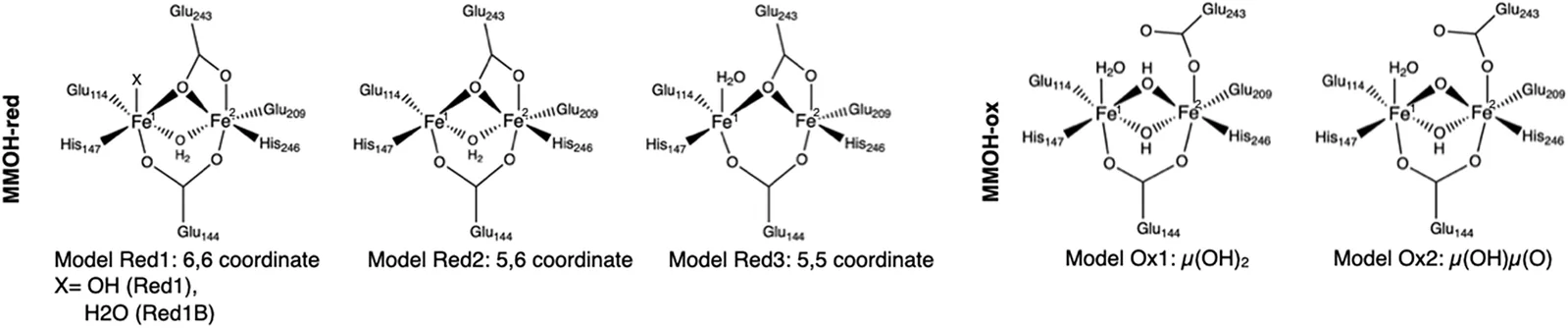
Depiction of local ligand environment for the calculated models of MMOH-red and ox both with and without MMOB. Models with MMOB are denoted in the text with a B (e.g., Ox1B).

In the case of MMOH-ox, a fully saturated 6,6 coordination sphere with one terminal and two bridging solvent ligands is widely accepted. Prior QM/MM calculations performed on sMMO, using a structure of MMOH alone (1MTY, 1.70 Å), identified a H_2_O–Fe-μ­(OH)­μ­(O)-Fe model (‘ox-3′ in ref 47, models Ox2 and Ox2B in the present work) as the most consistent with experimental data, out of eight tested models.^47^ This conclusion was based on the agreement with the 1MTY crystal structure, EXAFS bond lengths, Mössbauer, and previously published HERFD XAS data.^11,27,44,73,74^ Of the eight models, six resulted in Fe–Fe distances, Fe- terminal water bond lengths, and/or Mössbauer parameters inconsistent with experimental XRD structures and Mössbauer data. Notably, another model, H_2_O–Fe-μ­(OH)_2_-Fe (‘ox-2′ in ref 47, models Ox1 and Ox1B here), also displayed reasonable structural characteristics, including an appropriate Fe–Fe distance, and calculated Mössbauer parameters. However, this model was discarded in this prior work due because the calculated Fe K-edge XAS pre-edge intensities were inconsistent with previously published MMOH-ox:MMOB data.^47^ Since the experimental XAS used for comparison to QM/MM models has now been identified as representing the semimet form, we revisit this alternative structural motif in the present work. Accordingly, both experimentally plausible models (Figure 3 and Table 1) were selected for QM/MM studies in the presence and absence of MMOB.

Of the states presented here, MMOH-red:MMOB and MMOH-ox:MMOB, are most directly relevant to the catalytic cycle and thus, the most important to understand. A comparison of the experimental pre-edges of these two states with calculated models Red1B and Ox1B using the room temperature XFEL MMOH-ox:MMOB structure are shown in Figure 4. The relative IWAE shift upon reduction seen in the experimental spectra (−1.02 ± 0.10 eV) is well matched by the calculated spectra (−1.03 eV). The overall intensity of the model Red1B calculated pre-edge is somewhat higher than the model Ox1B calculated pre-edge (103%, 13.5 and 13.1), which differs from experiment as MMOH-red:MMOB has 80% (±13%) of the overall intensity of MMOH-ox:MMOB (12.7 and 15.8, respectively).

**Figure 4:**
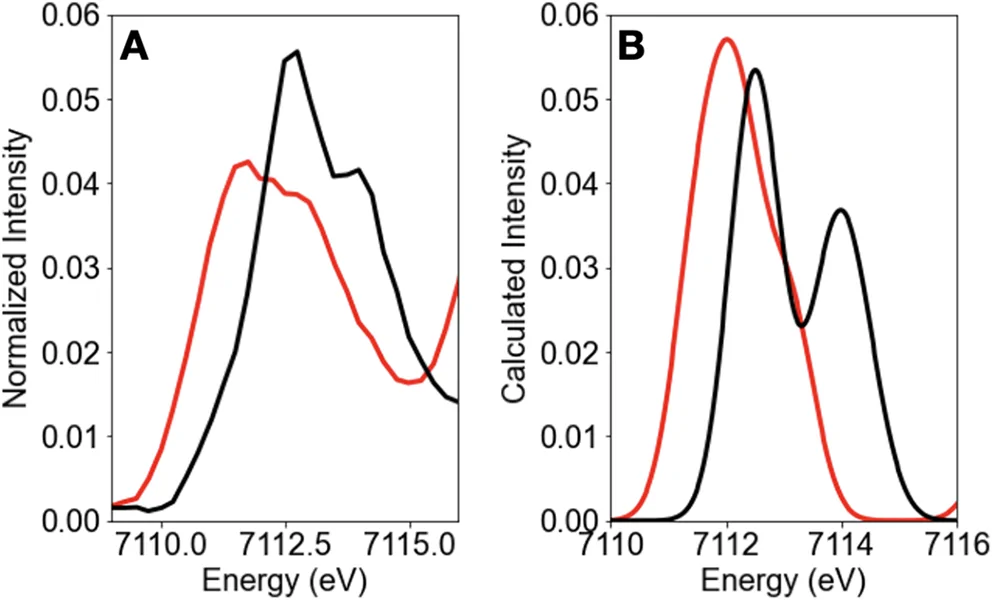
(A) Experimental HERFD XANES pre-edges of MMOH-red:MMOB (red line) and MMOH-ox:MMOB (black line) at 291 K. (B) Calculated pre-edges for models Red1B (red) and Ox1B (black).

The calculated pre-edge spectra of all MMOH-red and MMOH-red:MMOB models are shown in Figure 5, with the preferred MMOH-ox:MMOB model (Ox1B) and the highest intensity MMOH-ox­(B) model (Ox2B) shown for reference in Figure S18. The lower coordinated models Red2B and Red3B have significantly more intensity than all MMOH-ox and MMOH-ox:MMOB models (Figure S18). The calculated spectra of models Red2B and Red3B appear to have one broad feature, with the relative IWAE of the 5,6 coordinated active site slightly lower than that of the 5,5 coordinated active site. By contrast, the 6,6 coordinated active site of model Red1B is significantly lower in intensity and has two clear features, a peak at 7112.1 eV and a slight shoulder at 7113.0 eV. In the case of MMOH-red alone, the closest reasonable intensity remains the 6,6 coordinated model Red1, which has an overall pre-edge area and IWAE within the error of the experiment. As the 6,6-coordinate models Red1 and Red1B have intensities best in line with the experimental MMOH-red and MMOH-red:MMOB data, we assign both states as 6,6 coordinate.

**Figure 5:**
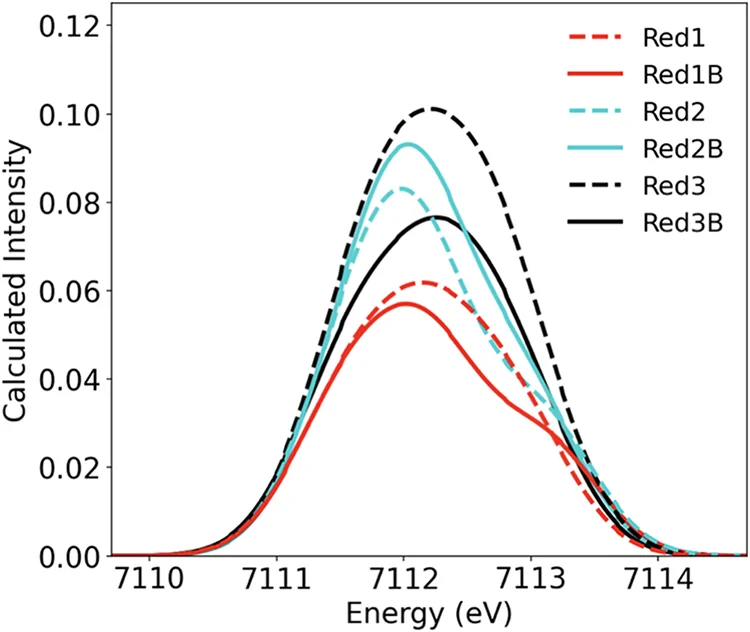
Calculated pre-edges for MMOH-red:MMOB (solid lines) and MMOH-red alone (dashed lines).

TD-DFT calculations typically break down for continuum transitions, which makes them unsuitable for computing changes in the rising edge. However, some insight into the whiteline modulations of MMOH-red and MMOH-red:MMOB can be obtained via comparison to molecular complexes. A comparison to three octahedral iron sites, previously published in Westre et al. (Figure 6),^72^ show similar trends between the more rigorously octahedral [Fe­(H_2_O)_6_]^2+^ and [Fe­(im)_6_]^2+^ and the distorted octahedral iron dimer, [Fe_2_(salmp)_2_] (where salmp = [2-[bis­(salicylidenamino)­methyl]-phenolate]^3–^) as is seen between MMOH-red and MMOH-red:MMOB. Octahedral continuous shape measurement (CShM)^71^ shows a clear trend of octahedral character [Fe­(H_2_O)_6_]^2+^ > [Fe­(im)_6_]^2+^ > [Fe_2_(salmp)_2_] that is commensurate with whiteline intensity. As such, we suggest that the whiteline shape is sensitive to distortions in geometry within a formally octahedral setting, where distortion from true octahedral results in loss of degeneracy between the 4p states and thus a broader, less intense transition. CShM analysis of the crystal structures of MMOH-red:MMOB at room temperature (6YDI) and of frozen MMOH-red alone (6VK7) show decreasing octahedral character upon MMOB binding of 31% (from 1.24 to 1.63, where values closest to 0 are most octahedral) at the Fe1 site (left in Figure 3) and of 2.5% (3.23 to 3.31) at the more distorted Fe2 site. As such, HERFD XAS shows clear spectral differences upon MMOB binding and subsequent minor perturbations of the ligand field that make it an excellent measure of relative geometry for similar systems. This analysis may hint at the distortion of local site symmetry of MMOH-red upon MMOB binding which could in turn enable subsequent ligand loss and molecular oxygen binding.

**Figure 6:**
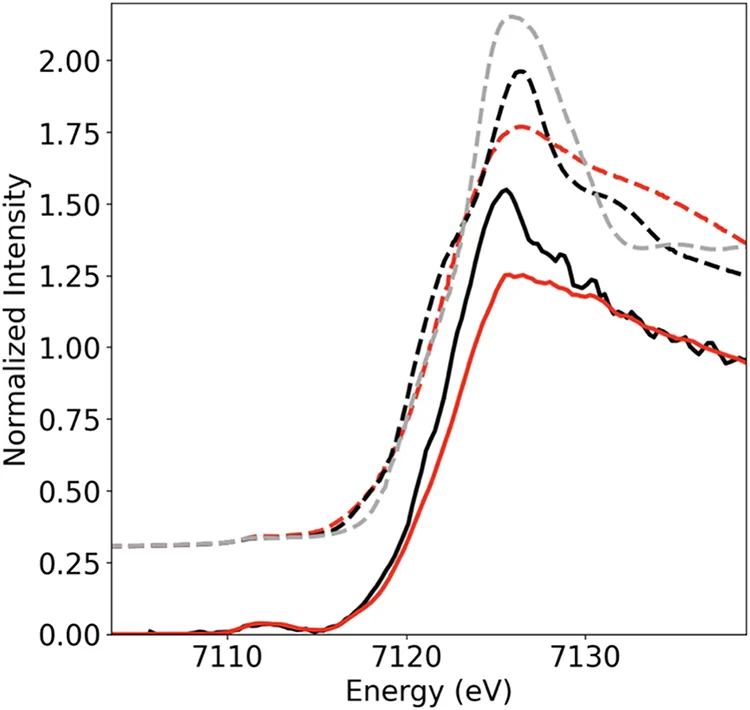
HERFD XAS at pH 7.0 of MMOH-red alone (black) and with MMOB (red) collected at 291 K shown in solid lines. Previously published XAS from Westre et al. of [Fe­(imidazole)_*6*_Cl_*2*_] (in black), [Fe­(H_*2*_O)_*6*_]­[(NH_4_)_*2*_] (in gray) and [Fe­(salmp)_*2*_] (in red) shown in dashed lines above, reproduced from ref 70, copyright 1996 American Chemical Society. Further characterization of [Fe­(salmp)_*2*_] is found in ref 70.

A comparison between the experimental pre-edges of the MMOH-ox states and calculated MMOH-ox models is shown in Figure 7. The calculated spectrum of model Ox1B has a first peak at 7112.5 (7.4) and a less intense feature at 7114.0 (5.8). The IWAE of both pre-edge features of the calculated model Ox1B spectrum are in excellent agreement with the experimental MMOH-ox:MMOB at pH 7 pre-edge, and the trend of having a higher intensity, lower energy feature is replicated. This is not the case for model Ox2B, which consists of 3 features (a shoulder at 7112.6 eV, a peak at 7113.3 eV, and a high intensity peak at 7114.6 eV) and thus has a higher IWAE of 7113.5 eV. The trend between the models Ox1B and Ox2B captures the redistribution of pre-edge intensity to higher energy seen with increasing pH from 7.0 to 7.5 for MMOH-ox:MMOB experimentally.

**Figure 7:**
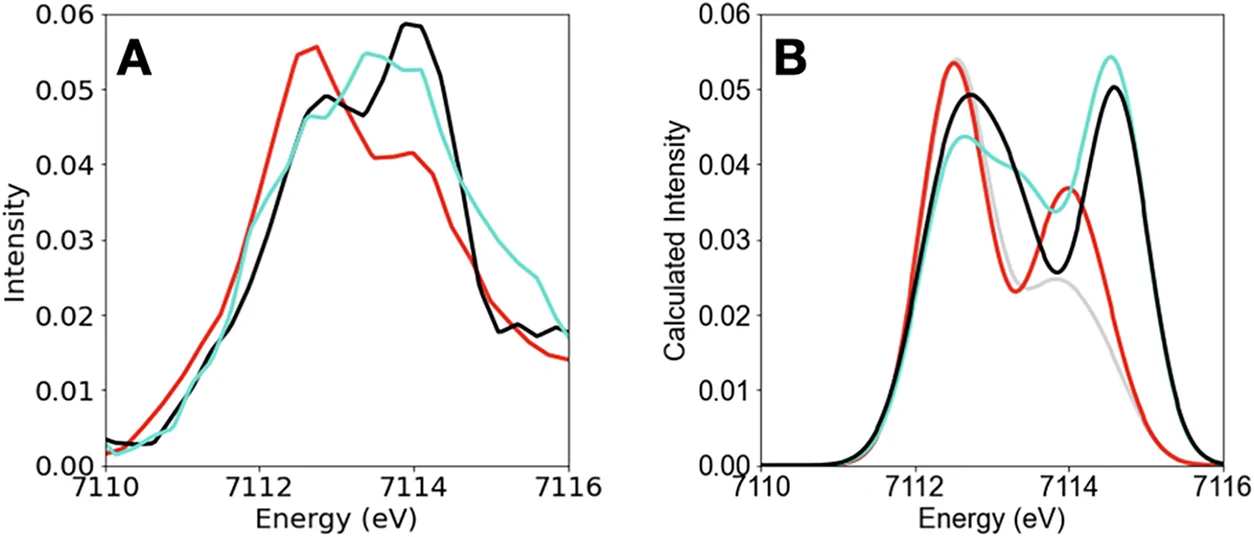
(A) Experimental pre-edges of MMOH-ox:MMOB at pH 7­(red), MMOH-ox alone at pH 7 (black), and MMOH-ox:MMOB at pH 7.5 (light blue). (B) Calculated pre-edges of Ox1B (red), Ox2B (light blue), Ox1 (light gray) and Ox2 (black).

In the absence of MMOB, model Ox1 has a high intensity feature at 7112.5 eV (7.6) and a low intensity shoulder at 7114.2 eV (4.3). Model Ox2 has similar intensity to lower energy, but the highest energy transitions (7114.6 eV) gain significant intensity compared to model Ox1 (from 1.6 to 6.7), resulting in an increase in overall intensity. As such, model Ox2 best replicates the relative intensity of features seen in the experimental MMOH-ox spectrum in the absence of MMOB. The best matches to experiment appear to be model Ox1B for MMOH-ox:MMOB at pH 7 and the deprotonated model Ox2B at pH 7.5, and the deprotonated model Ox2 for MMOH-ox alone at pH 7.0. As such, MMOB binding appears to either induce a protonation event at the active site or result in an analogous structural modification.

While analysis of the rather large 3.5 eV edge shift seen for MMOH-ox states compared to MMOH-red states via TD-DFT calculations suffers from the same issues regarding continuum transitions, comparison with diferric model complexes from Castillo et al. reveals the MMOH-ox and MMOH-ox:MMOB rising edge energies are quite similar to that of the diferric bis-μ–OH model (Figure S19), as well as to several of the Westre et al. molecular iron­(III) complexes, including the hexaaqua ferric salt.^27,72^ Additionally, the local geometry of both irons in the MMOH-ox states are significantly more octahedral than their counterparts in MMOH-red states (MMOH-ox:MMOB has an octahedral CShM of 0.8 for Fe1 and 1.5 for Fe2, compared to 1.6 and 3.3, respectively, in MMOH-red:MMOB), likely as a result of the glutamate shift that occurs upon reduction. These distortions modulate the ligand field and result in a larger edge shift than may be expected for a one unit change in oxidation state.

## Discussion

### Electronic and Structural Description of MMOH Active States

Analysis of the HERFD XAS data for MMOH-ox and MMOH-red supports assignment of a 6,6-coordinate geometry at the diiron active site of MMOH, irrespective of MMOB binding. For MMOH-ox alone, the active site structure is most consistent with a model that has one bridging oxo, one bridging hydroxide ligand, and a terminal water ligand, as previously assigned by Schulz et al.^47^ For the MMOH-ox:MMOB adduct, the correlation of spectroscopy and theory are most consistent with a model with two bridging hydroxide ligands and a terminal water ligand at pH 7. These models are also in agreement with existing crystallographic data. Further, without a change in protonation state, no model set matches the experimentally seen trend in the pre-edge upon MMOB binding at pH 7 (Figure S20). Upon increasing the pH, distinct changes in the pre-edge region of the MMOH-ox:MMOB adduct are observed, consistent with deprotonation at the active site and also analogous to the pre-edge signature of MMOH-ox alone. While the determination of the relative percentage of species in solution at pH 7 and pH 7.5 would require additional data, this pH dependent modulation in the experimental pre-edge is consistent with the TD-DFT calculated pre-edge shift seen between the protonated and deprotonated models.

A similar trend is noted for the reduced states, where the MMOH-red model lacking MMOB exhibits one less proton at the active site of the optimized structure relative to MMOH-red:MMOB. Although signal-to-noise limitations in the MMOH-red data at 291 K preclude definitive protonation state assignment, the overall trends are consistent with two proton-coupled electron transfer (PCET) events during the MMOH-ox to MMOH-red transition. Importantly, the calculated models preserve a neutral charge at the active site for both MMOH-ox:MMOB and MMOH-red:MMOB, in contrast to a net −1 charge for the unbound forms. Room temperature XFEL structures have previously identified a solvent-filled cavity adjacent to the active site containing an extensive hydrogen-bonding network that is modulated by MMOB binding. This cavity provides a plausible pathway for proton transfer to the diiron center.^16^ Thus, while the present data do not uniquely define the protonation states or populations of individual species in solution, the combined spectroscopic and computational results support a role for MMOB in modulating the local protonation environment and electrostatics of the diiron center.^2,75,76^

### Reevaluating the Coordination Number of MMOH-Red

The assignment of MMOH-red as a coordinatively unsaturated (5,5-coordinate) species has long underpinned mechanistic proposals for sMMO, invoking such unsaturation as a prerequisite for O_2_ activation.^34,35^ These assignments, initially based on VTVH MCD and ligand field analyses of model complexes, were made prior to the availability of MMOH-red crystal structures.^19^ Subsequent spectroscopic studies did not readdress coordination number explicitly and largely relied on structurally symmetric models.^17,18^ Further, we note that a low energy d-d band predicted at 4930 cm^–1^ based on the calculated 5-coordinate ligand field transition energies in MMOH-red was in fact not observed experimentally. Subsequent crystallographic data revealed substantial asymmetry at the active site, with Fe–O distances ranging from 1.94 to 2.51 Å and a compressed O–Fe–O angle of 57.7° arising from Glu243 coordination, which results in local site symmetries at the irons that deviate dramatically from the 6-coordinate distorted octahedral models discussed by Solomon and coworkers.^16–19,23,77^

QM/MM-derived d-d transition energies for all MMOH-red:MMOB models were calculated using TD-DFT, and the four spin-allowed d-d transitions for each iron in the active site are depicted in Figure S21 for the three models. While clear modulations in the individual d-d transitions are observed across the series, the average ligand field energies are approximately the same (6903, 7042, 7096 cm^–1^, for 5,5, 5,6 and 6,6-coordinate, respectively), suggesting the 1s-3d pre-edge energy is not expected to differ significantly between the models, as seen in Figure 5. Figure S21 also depicts the previously reported MCD fit values for three of the d-d bands, which were observed in the 6000 to 10,000 cm^–1^ region. While these data were used to assign a 5,5-coordinate environment, the current study suggests that alternate fits to the broad envelope of MCD transitions should also be considered. A more conclusive assignment would be enabled by experimental data for the d-d transitions below 5000 cm^–1^, which while challenging optically, would be accessible by recently reported 2p3d RIXS (resonant inelastic X-ray spectroscopy) approaches.^78^ In the current study, however, both the experimental resolution and the 1s core hole lifetime broadening limit the present interpretation to the average ligand field energy. We hypothesize that the average ligand field of MMOH-red:MMOB is not strongly modulated by changes in coordination number because the ligand field is dominated by interactions with the most strongly bound histidine and glutamate residues (bond lengths 1.9–2.15 Å), and the addition of a relatively weakly donating water (∼2.3 Å Fe–O bond length) thus has limited impact on the overall ligand field. Notably, however, the addition of a sixth ligand, even one with weakly donating character, does result in a higher degree of symmetry at the iron center and thus lowers the degree of 3d-4p mixing. As pre-edge intensity reflects the degree of 3d-4p mixing, HERFD XAS pre-edges provide greater sensitivity to such nuanced geometries, and dramatic differences in pre-edge intensity occur with changes in coordination number of the models. The high intensity pre-edges of the calculated 5,5 and 5,6-coordinate models shown here are incongruous with experimental data. These results support a 6,6-coordinate ground state for MMOH-red and MMOH-red:MMOB, consistent with available crystallographic data.

While this finding challenges the previously published view that nonheme diiron enzymes initiate catalysis in a coordinatively unsaturated state,^18,34,79^ it does not preclude transient unsaturation during turnover. As the generation of the substrate-reactive Q intermediate is possible in absence of the hydrocarbon substrate, sMMO clearly does not rely on a mechanism where the substrate opens the coordination sphere for oxygen interaction, as has been suggested for nonheme mononuclear iron enzymes.^80^ One possibility is that following the initial irreversible oxygen binding step (intermediate O), an active site-bound water ligand is displaced to afford oxygen access, thus generating intermediate P*.^81^ The transition between intermediate O and intermediate P* may represent the moment in which unsaturation is generated and oxygen becomes properly positioned for subsequent activation steps leading to the diferric peroxo (P) and the Fe­(IV)­Fe­(IV) (Q) intermediates.

### Cryogenic Perturbations to Electronic Structure

Cryocooling induced alterations in protein structure and electronic configuration are increasingly recognized as a critical challenge for both structural and spectroscopic studies.^31,33,82^ In this work, HERFD XAS spectra of MMOH-red:MMOB reveal clear changes to the edge region upon cryocooling. Although similar perturbations are not obvious for MMOH-ox and MMOH-ox:MMOB, higher noise in the low temperature spectra may obscure subtle effects. In previous work from our group, Cutsail et al. focused on identifying and correcting for background iron contamination in PFY XAS spectra of what was then believed to be MMOH-ox:MMOB.^44^ Based on the findings of the present study, we now recognize that the sample in question likely corresponds to the semimet form. Nevertheless, upon revisiting those data, we observe that the two freezing methods used for preparing MMOH-ox:MMOB (semimet) produced distinct differences in whiteline shape, which cannot be explained solely by background iron contamination (Figures S22 and S23). This provides further evidence that the method of freezing can significantly influence the electronic structure observed by XAS.

Previously reported PFY XAS data of MMOH-red and MMOH-red:MMOB from *Mc* Bath display trends that parallel the 291 K data presented here, despite having been collected under cryogenic conditions.^26^ Resolving this discrepancy will require further studies beyond the scope of the present work to determine whether differences in solution composition or protein strain influence the spectroscopic signatures and temperature dependence of sMMO. These findings suggest that, without direct comparison to room temperature data, frozen state investigations of metalloenzyme electronic structure may not reliably reflect catalytically relevant states. In the case of sMMO, the pronounced cryogenic sensitivity of MMOH-red:MMOB, which initiates the catalytic cycle, raises the possibility that other intermediates in the cycle may also be differentially affected by sample freezing.

### Radiation Sensitivity of MMOH-ox

This study provides the first radiation damage free HERFD XAS spectra of MMOH-ox and MMOH-ox:MMOB. In previous studies, regardless of the presence of MMOB, cumulative X-ray exposure likely led to partial reduction to Fe­(III)/Fe­(II) “semimet” states. The only exception appears to be the work of DeWitt et al., who reported PFY XAS on MMOH-ox in the absence of MMOB.^83^ Interestingly, the first observable damage event in MMOH-ox occurred at a similar dose under both cryogenic and room temperature conditions and was 3 orders of magnitude below the Henderson limit.^28^ This suggests that the initial radiative reduction event is not mitigated by temperature dependent radical diffusion. This appears to be consistent with the fact that previous studies have shown a shift in the edge position of MMOH-ox to occur without coupled changes in the EXAFS regime,^26,84^ potentially suggesting an electronic reduction has occurred that is not coupled to geometric relaxation.^85,86^ We note that previous studies have shown an inequivalence between chemical vs beam-induced reduction of metalloproteins, which further supports such a hypothesis.

### pH-Dependent Electronic Structure Modulation

The clear change in the pre-edge spectrum of MMOH-ox:MMOB between pH 7 and 7.5 suggests that the measurements were collected near the p*K*_a_ of a protonation equilibrium that influences the structure of the active site. As a result, one or both samples likely contain a mixture of protonation states. The observed increase in pre-edge energy at higher pH is consistent with the calculated trend. By contrast, the pH 7 MMOH-ox alone species is more consistent with the deprotonated model Ox2.

This work represents direct evidence of the impact of MMOB binding on the active site electronic structure of MMOH-ox. The changes to the pre-edge of MMOH-ox upon MMOB binding indicate that MMOB induces either a protonation (or a protonation-like effect) at one of the bridging oxo ligands. Given the current work and previous crystallographic studies showing MMOB binding modulates the cavity adjacent to the active site,^16,23^ evidence supports the organization of a hydrogen bonding network in the active site adjacent cavity upon MMOB binding which may serve as a proton source during turnover.

## Conclusion

The application of room temperature HERFD XAS, enabled by low-dose, sample efficient laminar flow sample delivery, has allowed for the first acquisition of radiation damage-free spectra of MMOH-ox and MMOH-ox:MMOB. These measurements offer new insights into the coordination environment and electronic structure of soluble methane monooxygenase (sMMO) under catalytically relevant conditions.

The data presented here support a 6,6-coordinate geometry for all characterized MMOH states, including those previously proposed to be coordinatively unsaturated. It has been previously proposed that the ferrous active sites of soluble methane monooxygenase (sMMO), ribonucleotide reductase (RNR), and hemerythrin (Hr) are coordinatively unsaturated, providing potential sites for small molecule binding at the metal center.^18,35,79^ In sMMO, however, it has long been established that exogenous ligands, including the hydrocarbon substrate, do not bind directly to the reduced Fe­(II) center, raising questions about how oxygen access is achieved. Importantly, Solomon and co-workers have also noted that the compact nature of the diferrous active site in MMOH, together with an increased coordination number at the iron centers, can introduce sufficient steric hindrance and charge donation to disfavor binding of small anionic ligands.^18^ In addition, solvent molecules and extended hydrogen-bonding networks at the iron cluster sites may further inhibit anion binding either sterically or through electrostatic effects associated with a partially negative solvation environment. Our current results, which support a six-coordinate Fe­(II) site in MMOH-red, help reconcile these perspectives by indicating that “coordinative unsaturation” should not be interpreted as a static or binding-accessible property of the resting Fe­(II) state. Instead, oxygen reactivity may be more appropriately understood as arising from a protein-gated (or in some cases substrate-gated) reorganization of the coordination sphere that generates an oxygen-accessible configuration along the reaction coordinate. This view is supported by the characterization of the diferrous, oxygen-bound sMMO intermediate P*, which provides evidence for such a gated entry pathway.^81^ In this framework, molecular oxygen activation in sMMO is governed by a dynamic restructuring of the diiron active site that precedes the formation of high-valent intermediates such as Q, rather than originating from a pre-existing coordinatively unsaturated Fe­(II) center.

Additionally, this work shows that HERFD XAS is sensitive to subtle pH-dependent changes in the protonation state of the active site, revealing a pH-sensitive group with a p*K*_a_ in the physiological range that likely governs access to the intermediates P and Q. Both HERFD XAS and multiscale QM/MM calculations indicate that MMOB binding modulates both the electronic structure and protonation state, reinforcing its essential role in enabling catalytic turnover. Beyond sMMO specific structural insights, these findings underscore the potential limitations of cryogenic methodologies in resolving native enzyme reactivity. Cryocooling and radiation damage introduce distortions that may obscure key intermediates or misrepresent redox states. The approach employed here effectively mitigates these artifacts, offering a path forward for time-resolved or pH-dependent spectroscopies of redox-active metalloenzymes under ambient conditions. Taken together, these results provide a refined structural and electronic framework for sMMO function and highlight the broader potential of HERFD XAS combined with microfluidics for capturing catalytically relevant states in complex metalloproteins.

## Supplementary Material



## Data Availability

The data that supports the findings of this study are available on EDMOND under the link below: 10.17617/3.0FV1UN.

## Notes

Extended methods for flow cells, microfluidic mixer and sample delivery, radiation damage studies on MMOH-ox:MMOB, and computational methodology; flow cell schematics; fluidics for anaerobic data collection; fits of experimental HERFD XAS pre-edges; bond lengths of MMOH-red models; reproduction of raw data from Cutsail 2018; flow conditions; experimental fit parameters of HERFD XAS pre-edges with uncertainties (PDF)

## ∇

Institute for Integrated Catalysis, Pacific Northwest National Laboratory, Richland, Washington 99354, United States

## ○

Department of Bioengineering and Therapeutic Sciences, University of California San Francisco, San Francisco, California 94158, United States
