# Supplementary material for: High-Resolution X‑ray Absorption Spectroscopy of Soluble Methane Monooxygenase: Room Temperature and Cryogenic Insights into Active Site Electronic Structure Modulation
Source: J Am Chem Soc. 2026 Oct 15;148(42):45749–62. doi: 10.1021/jacs.6c13395 (PMC13721151; doi:10.1021/jacs.6c13395)
Supplement: Supplementary file 1 [file ja6c13395_si_001.pdf]

High-Resolution X-ray Absorption Spectroscopy of Soluble Methane Monooxygenase: Room Temperature and Cryogenic Insights into Active Site Electronic Structure Modulation

Olivia McCubbin Stepanic,[A] Sinjini Bhattacharjee,[B+] Christopher J. Pollock,[C] Kara A. Zielinski,[D++] Rahul Banerjee,[E,F] Derek B. Rice,[A] Kushal Sengupta,[A] John D. Lipscomb,[E] Dimitrios A. Pantazis,[B] Frank Neese,[B] Lois Pollack,[C] Serena DeBeer.[A*]

A- Max Planck Institute for Chemical Energy Conversion, Mülheim an der Ruhr, Germany 45470

B- Max-Planck-Institut für Kohlenforschung, Mülheim an der Ruhr, Germany 45470

+-Current Affiliation- Institute for Integrated Catalysis, Pacific Northwest National Laboratory, Richland, WA, USA 99354

C- Cornell High Energy Synchrotron Source, Wilson Laboratory, Cornell University, Ithaca, NY, USA 14853

D- School of Applied Engineering and Physics, Cornell University, Ithaca, NY, USA 14853

E- Department of Biochemistry, Molecular Biology, and Biophysics, University of Minnesota, Minneapolis, MN, USA 55455

F- Department of Chemistry and Biochemistry, Auburn University, Auburn, AL, USA 36849

++-Current Affiliation- Department of Bioengineering and Therapeutic Sciences, University of California San Francisco, San Francisco, CA, USA 94158

* serena.debeer@cec.mpg.de

SUPPORTING INFORMATION

Table of Contents

	Page
**Microfluidic Flow Cells and Sample Delivery**	4-6
Figure S1. Flow cell schematics.	4
Figure S2. Fluidics for ambient data collection.	5
Figure S3. Fluidics for anaerobic data collection.	5
Table S1. Flow conditions.	6
Figure S4. Example of UV/vis spectra from MMOH-ox:MMOB, collected to monitor flow stability.	6
**MMOH-ox:MMOB radiation damage studies**	7-9
Figure S5. Area normalized HERFD XANES spectra of sMMOH-ox: MMOB at various doses and temperatures.	8
Figure S6. Area normalized scans of MMOH-ox:MMOB at a range of doses exemplifying overlays and damage regime.	9
Figure S7. Unsmoothed HERFD XANES data.	10
Figure S8. Fits of experimental HERFD XAS pre-edges.	11
**Computational Methodology**	12-18
Figure s9. Depiction of the complete setup of MMOH:MMOB and MMOH alone solvated within the TIP3P water box and counterions.	12
Fig. S10 QM cluster model of sMMO.	13
Figure S11. Depiction of equilibration dynamics.	14
Figure S12. Overlay of crystal structure and last frame of equilibration simulations.	14
Figure S13. The final QM region used in this work, indicating specific H-bonding interactions around the active site.	15
Figure S14. Bond lengths of MMOH-ox models.	16
Figure S15. Bond lengths of MMOH-red models.	16
Table S2. Mulliken spin populations of Fe centers in each QM/MM model	17
**Additional Data**	19-26

Table S3. Experimental fit parameters of HERFD XAS pre-edges with uncertainties.	19
Figure S16. Select pre-edge spectra overlays with standard error.	20
Figure S17. Difference spectrum of MMOH-ox:MMOB pre-edge with the pH 7.5 data subtracted from pH 7	21
Figure S18. Comparison of all calculated MMOH-red and MMOH-red:MMOB models with MMOH-ox models Ox1B and Ox2B.	21
Figure S19. Comparison of HERFD XAS of diferric models from Castillo 2017 with previously published MMOH-ox:MMOB (now assigned semimet) and the current MMOH-ox:MMOB at 291 K.	22
Figure S20. Comparison of experimental HERFD pre-edges of MMOH-ox (black) and MMOH-ox:MMOB (red) at pH 7 and all viable pairings of calculated pre-edges for MMOH-ox (black) and MMOH-ox:MMOB (red) models.	22
Figure S21. TDDFT calculated d-d transition energies of the MMOH-red:MMOB QM/MM models.	23
Figure S22. Reproduction of raw data from Cutsail 2018 Figure 8.	24
Figure S23. Comparison of Cutsail 2018 powder MMOHox:MMOB data contaminated with various amounts of iron foil and frozen solution MMOHox:MMOB data	24

Microfluidic Flow Cells and Sample Delivery

The two flow cells used for data collection are shown in Figure S1. The Kenics flow cell was fabricated similarly to as previously described,[1] but different holders were used to make the quartz accessible from more angles. Additionally, Kapton tubing (MicroLumen Inc.) was used as a spacer between the Kenics support glass (556 µm ID, 600 µm OD, Hilgenberg GmbH) and the quartz observation region (800 µm ID, 840 µm OD, Hilgenberg GmbH). The co-flow flow cell was fabricated similarly to a previously published design.[2] The co-flow cell was used for all damage studies.

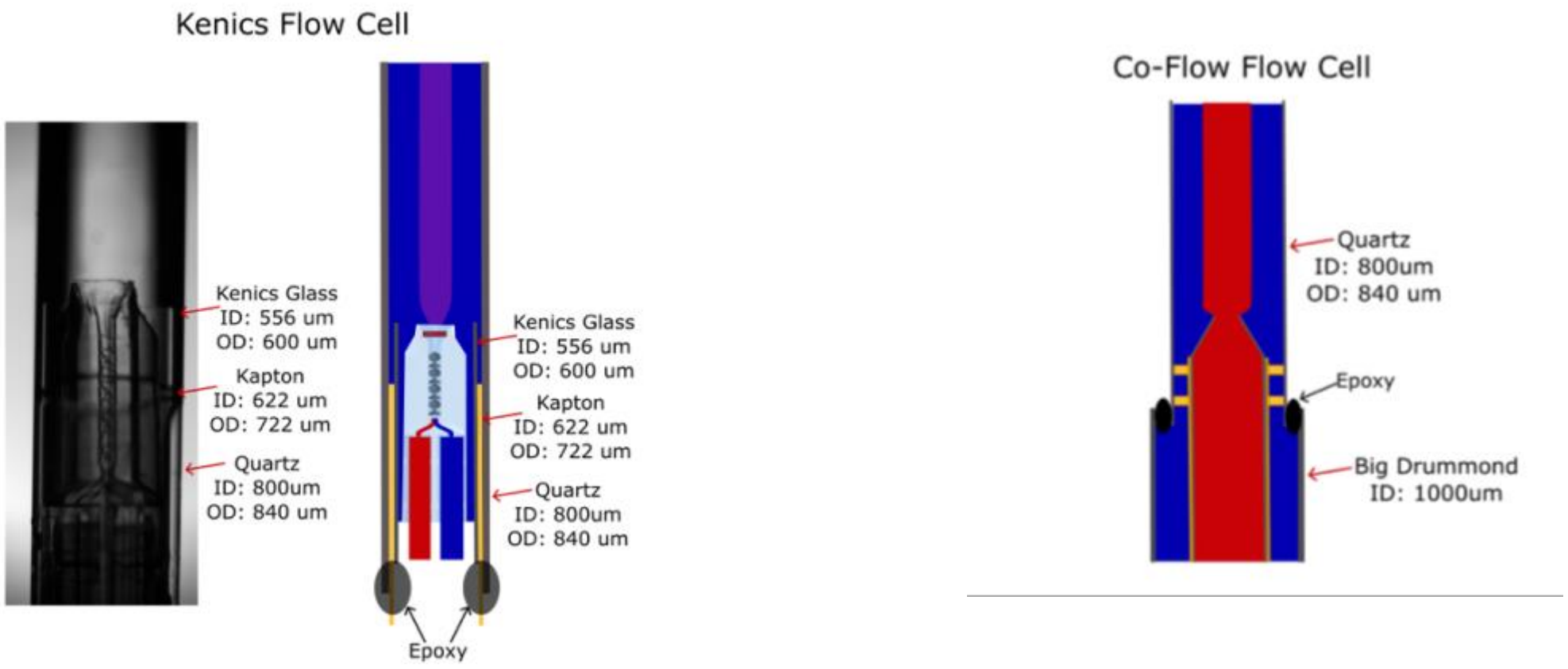

Figure S1. Flow cell schematics. Both the Kenics and co-flow flow cells are depicted. All components are labelled, including where the epoxy is applied to seal the devices.

The ambient and anaerobic fluidic schematics are shown in Figures S2 and S3, respectively. Both setups use the same components. In particular, the sheath is driven by the Elveflow OB1 flow controller and the sample and mixing lines use HPLC pumps. For the co-flow flow cell, the mixing buffer fluidics were not needed. Additional modifications were made for the anaerobic setup to keep everything oxygen free. Specifically, the HPLC pump reservoir, sample reservoir, and injection valve are all placed in a glove bag for the sample line as all components needed to be manipulated for sample delivery, and thus all had to be kept directly anaerobic. The mixing buffer line only needed the HPLC pump reservoir to be anaerobic as it flowed continuously. The HPLC pump and injection valve kept the buffer sufficiently anaerobic for the travel time from the HPLC to the flow cell.

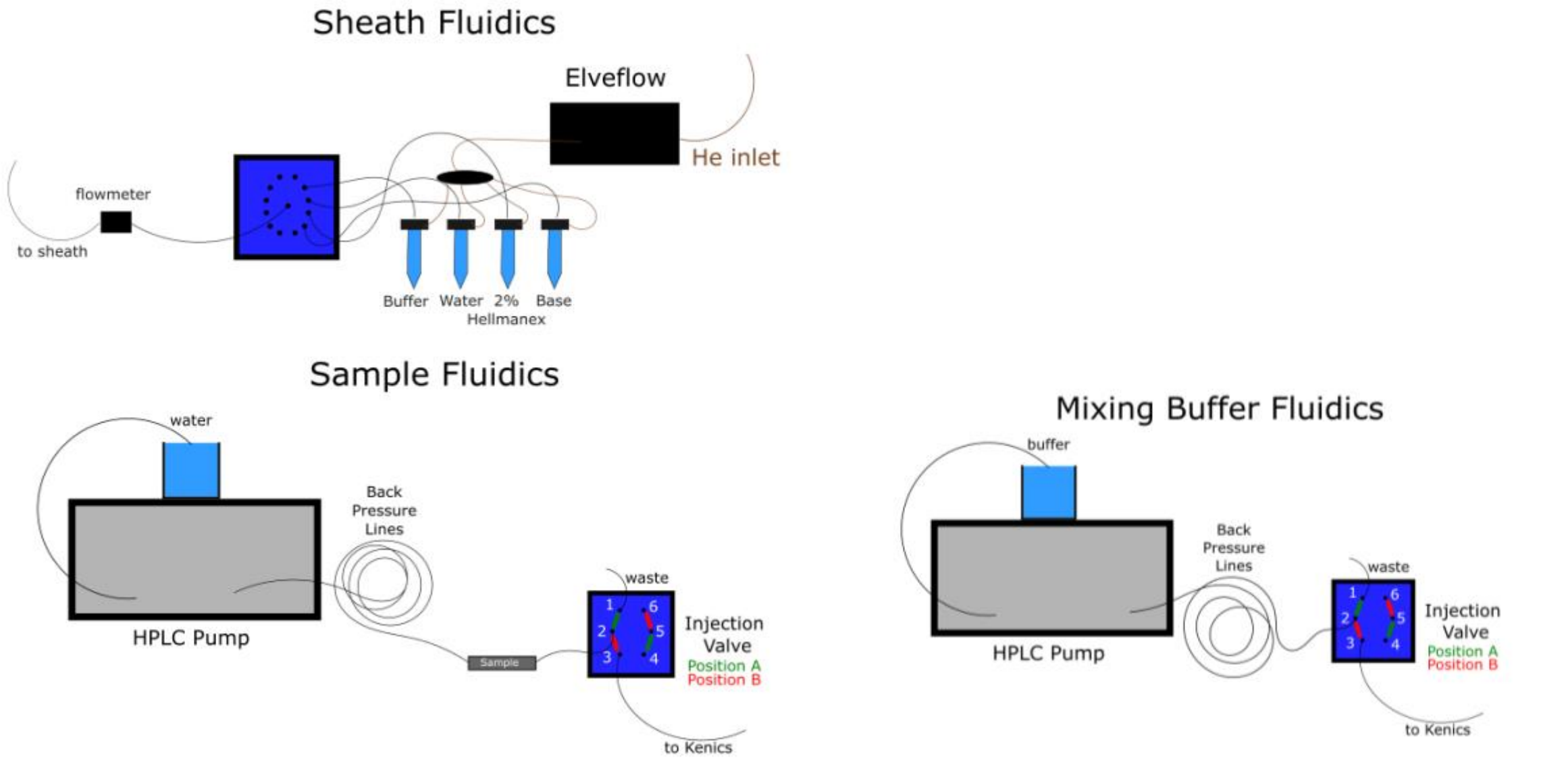

Figure S2. Fluidics for ambient data collection. The detailed flow paths for the sheath, sample, and mixing buffer lines are depicted. All components are kept at ambient conditions. For the sheath line, the He flow is depicted in brown and the fluid flow is depicted in black. A selector box was implemented for easy switching between species. For the sample and mixing buffer lines, 100 µm ID PEEK was used to increase the back pressure of the flow path to improve flow stability.

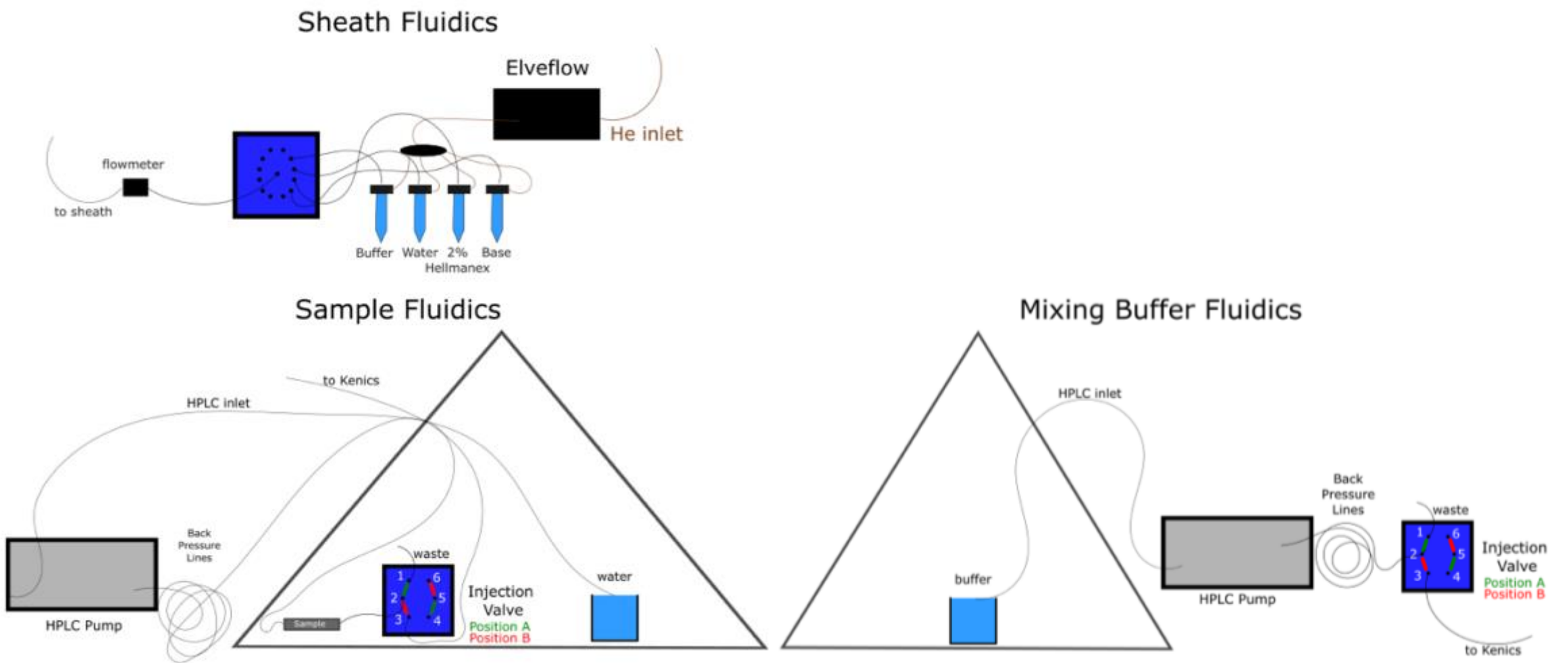

Figure S3. Fluidics for anaerobic data collection. The detailed flow paths for the sheath, sample, and mixing buffer lines are depicted. All components are kept at anaerobic conditions. For the sheath line, the He flow is depicted in brown and the fluid flow is depicted in black. Pressurizing with He was sufficient to make all buffers anaerobic. A selector box was implemented for easy switching between species. For the sample and mixing buffer lines, all components that needed to be manipulated during data collection were placed in glove bags. Two glove bags were used due to space constraints. Additionally, on both the sample and mixing buffer lines 100 µm ID PEEK was used to increase the back pressure of the flow path to improve flow stability.

Table S1. Flow conditions: For both the co-flow and Kenics flow cells, the flow rates were adjusted so that sample stream width was 400 μm, except for the 1.1 μL/min + 1 μL/min condition, which has a sample stream width of 450 μm. The total sample flow rate column reflects the flow rate for the co-flow flow cell. For the Kenics flow cell, each Kenics port had a flow rate of half the total sample flow.

Total Sample Flow Rate (μL/min)	Sheath Flow Rate (μL/min)	Average Velocity (mm/s)	Beam Residence Time (s)
12	15.5	1.596	0.251
8	10.2	1.058	0.378
6	7.8	0.796	0.503
4	5.2	0.531	0.753
2	2.6	0.265	1.509
1.1	1	0.115	3.478

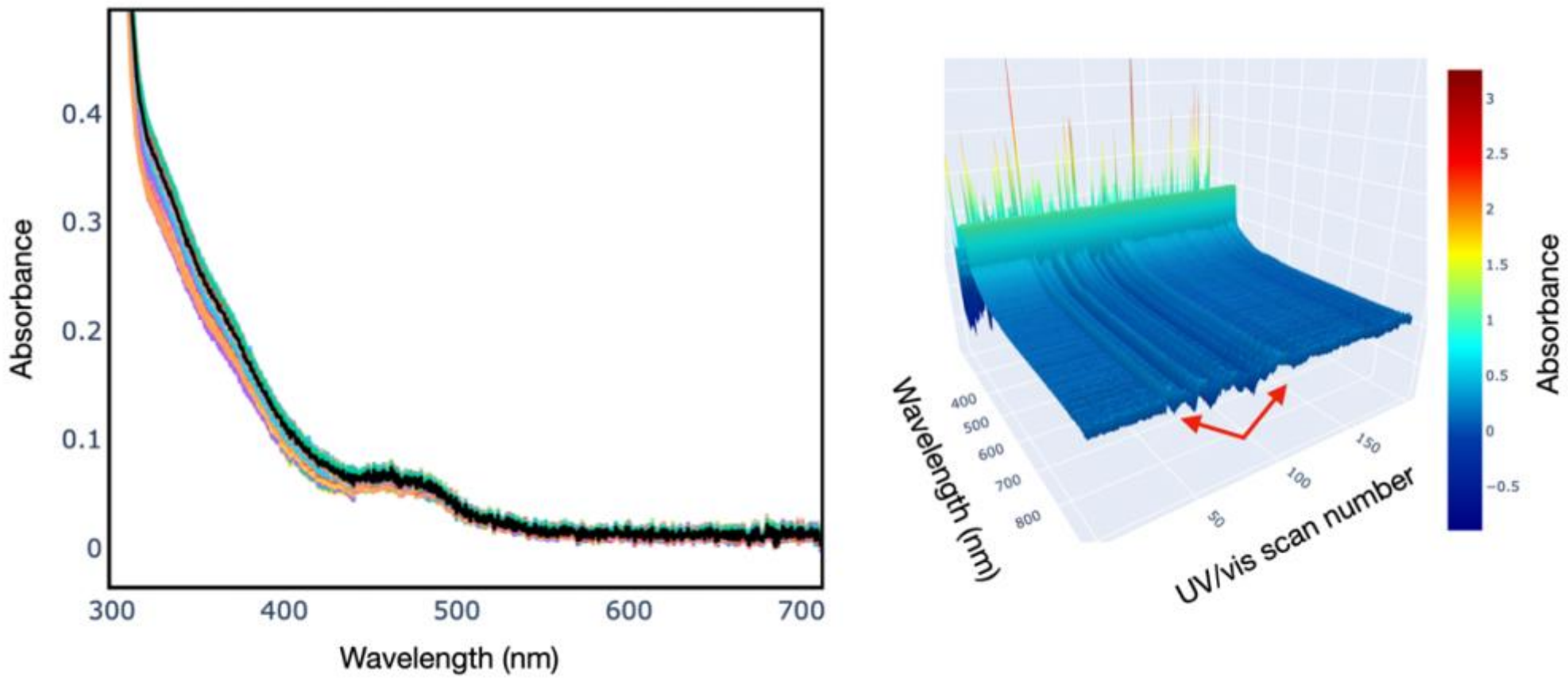

Figure S4. A. UV/vis spectra of MMOH-ox (and MMOR-ox, which contributes the features at 450 nm) collected during HERFD XAS measurement and suitably stable flow. B. An example of flow instability during measurement, manifesting as a change in the baseline absorbance, highlighted by the red arrows. HERFD XANES data collected during flow instability were discarded.

Microfluidic References

(1) Zielinski, K. A.; Katz, A. M.; Calvey, G. D.; Pabit, S. A.; Milano, S. K.; Aplin, C.; San Emeterio, J.; Cerione, R. A.; Pollack, L. Chaotic Advection Mixer for Capturing Transient States of Diverse Biological Macromolecular Systems with Time-Resolved Small-Angle X-Ray Scattering. *IUCrJ* **2023**, *10* (3), 363–375. https://doi.org/10.1107/S2052252523003482.
(2) Plumridge, A.; Katz, A. M.; Calvey, G. D.; Elber, R.; Kirmizialtin, S.; Pollack, L. Revealing the Distinct Folding Phases of an RNA Three-Helix Junction. *Nucleic Acids Res.* **2018**, *46* (14), 7354–7365. https://doi.org/10.1093/nar/gky363.

MMOH-ox:B radiation damage studies

The previously reported data (Castillo 2017) were collected with a beam spot ⅛ of the size of the data presented here and had significantly longer exposure times. For a quantitative comparison, a series of damage studies were performed with doses ranging from 1.8×10^5 to 5.7×10^8 J/kg at 30 K at the ID26 beamline in ESRF, doses ranging from 4.1×10^3 to 3.5×10^5 J/kg at 77 K at the PIPOXS beamline in CHESS, and doses ranging from 1.6×10^3 to 2.5×10^5 J/kg at 291 K at the PIPOXS beamline in CHESS, the results of which are summarized in Figure 5. Data collected at ESRF shows a clear shift in the rising edge to lower energy between ~4 x 10^6 J/kg and ~7 x 10^7 J/kg, and a plateau where spectra overlay between ~1 x 10^5 ph/mm^2 and ~2 x 10^6 J/kg. As damage assessments are typically performed by reducing the dose until two spectra of sufficiently different doses overlay, most damage assessments would stop in this range. Additionally, the spectrum with a dose of 1.8×10^5 J/kg was the average of five second scans on 101 spots with an attenuation of 97.4% of the beam and required about 25 minutes to collect a useful damage scan ranging from 7105 to 7135 eV (0.25 eV steps, sample exposure time: 8 min 25 s).

By contrast, the room temperature data were collected at doses ranging from 51.6×10^3 to 2.5×10^5 J/kg, which necessitated changing the mono crystals from Si(311) to Si(111) to exploit the additional ~5fold increase in incident flux, at the loss of some energetic resolution. Here, we see overlaying spectra at 1.1 and 2.3×10^3 J/kg, with some small damage starting to appear around 2×10^4 J/kg. From 2×10^4 to 2×10^5 J/kg, a clear damage event occurs. The spectrum with a dose of 2×10^5 J/kg was collected with no beam attenuation and the slowest possible flow (1.1 uL/min for sample and 1 uL/min for sheath) in the given setup.

To confirm that the damage events seen at room temperature on the order of 10^5 J/kg and at 30 K on the order of 10^7 J/kg are separate reduction events, data was collected under frozen conditions (77 K) at PIPOXS with the express purpose of matching the doses seen in the room temperature experiment. XAS scans at lowest collected doses had count rates on the order of 1 ct/s, and the resultant spectra took 14 hours each to collect. An estimated 24-48 hours would be required for a publication quality HERFD XAS spectrum of these samples. Here, a damage event again occurs in the range of 2×10^4 to 2×10^5 J/kg, consistent with the room temperature data. As such, MMOH-ox:MMOB has two distinct dose-dependent edge shifts which occur nearly two orders of magnitude of dose apart.

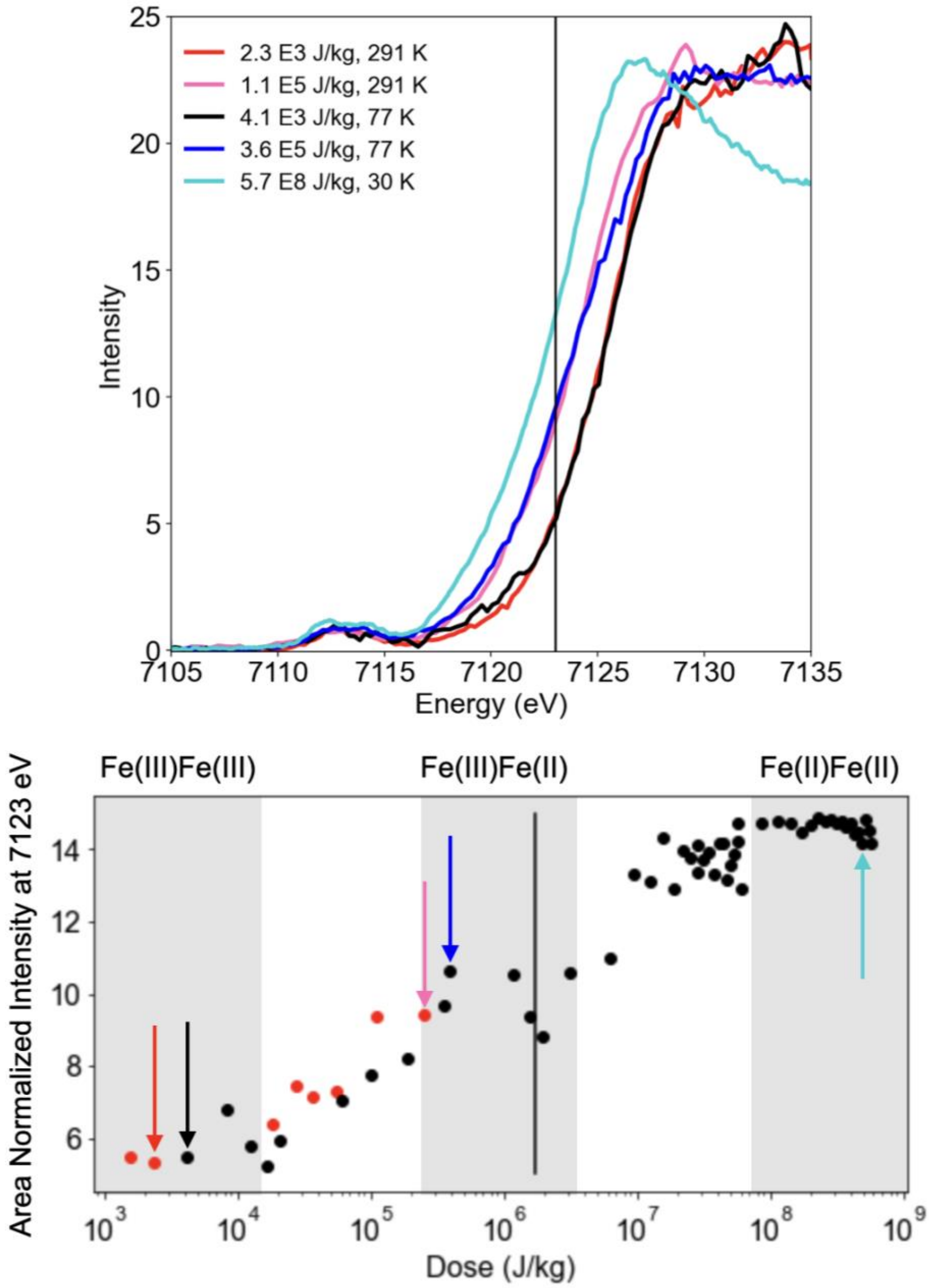

Figure S5. Top, area normalized HERFD XAS spectra of MMOH-ox:MMOB at various doses and temperatures. Below, a reproduction of Figure 2 from the main text, with color coded arrows highlighting the doses of the above spectra.

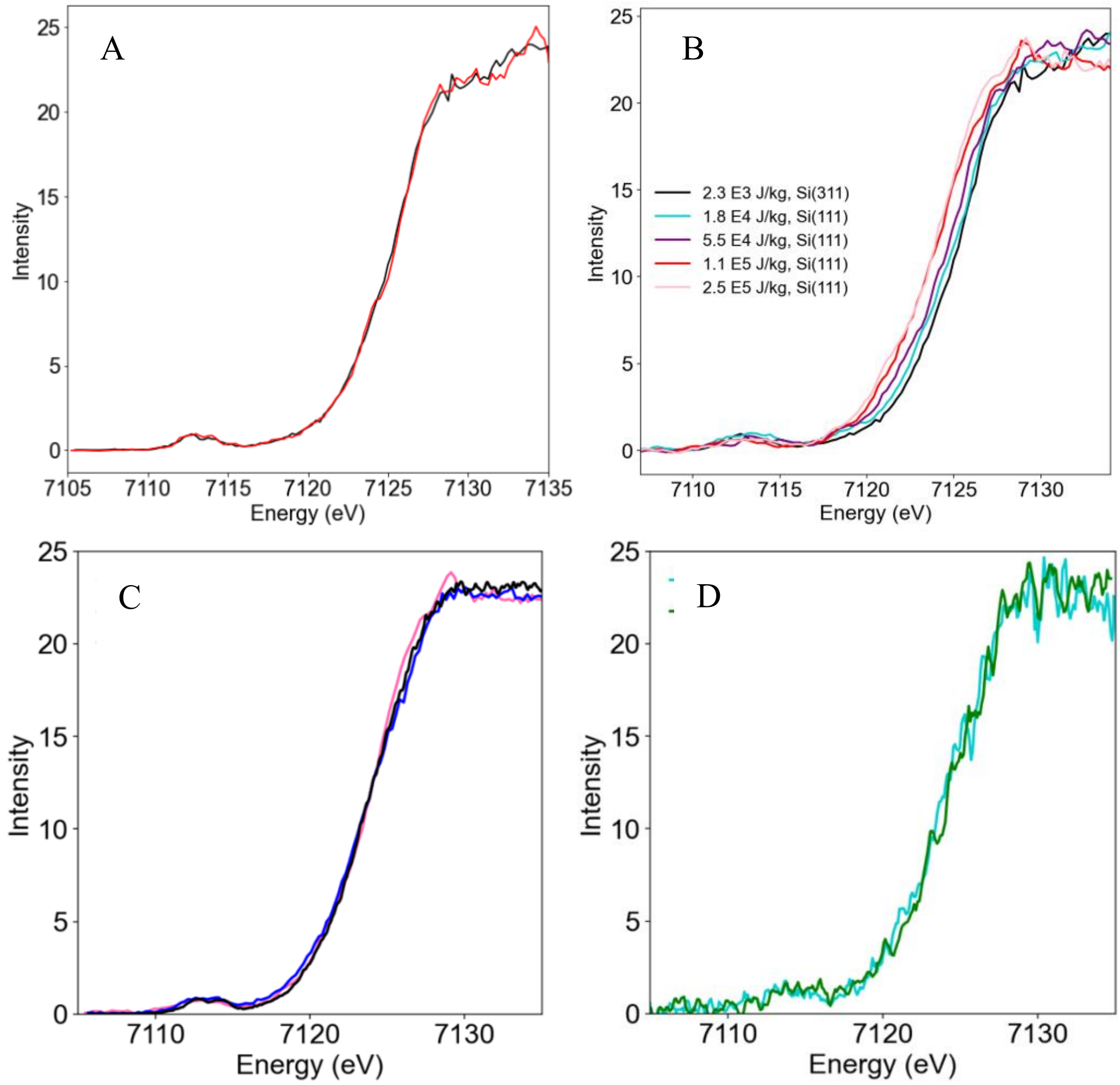

Figure S6. A. Area normalized scans of MMOH-ox:MMOB (291 K) at doses of 1.5x10^{3} J/kg (red) and 2.3x10^{3} J/kg (black) show clear overlay. B. A selection of area normalized scans of MMOH-ox:MMOB (291 K) at a range of doses from 2.3x10^{3} J/kg to 2.5x10^{5} J/kg illustrate the dependence of the edge position on dose between the Fe(III)Fe(III) state and the semimet plateau. C. Overlay of a room temperature semimet spectrum with a dose of 1.1 x 10^{5} J/kg (pink), a 77 K semimet spectrum with a dose of 3.6 x 10^{5} J/kg (blue) from CHESS data collection and the previously published data reproduced from [1], copyright 2017 American Chemical Society (in black). D. Overlay of semimet spectra from the ESRF damage study at doses of 1.4x10^{5} J/kg (dark green) and 1.1x10^{6} J/kg (light blue).

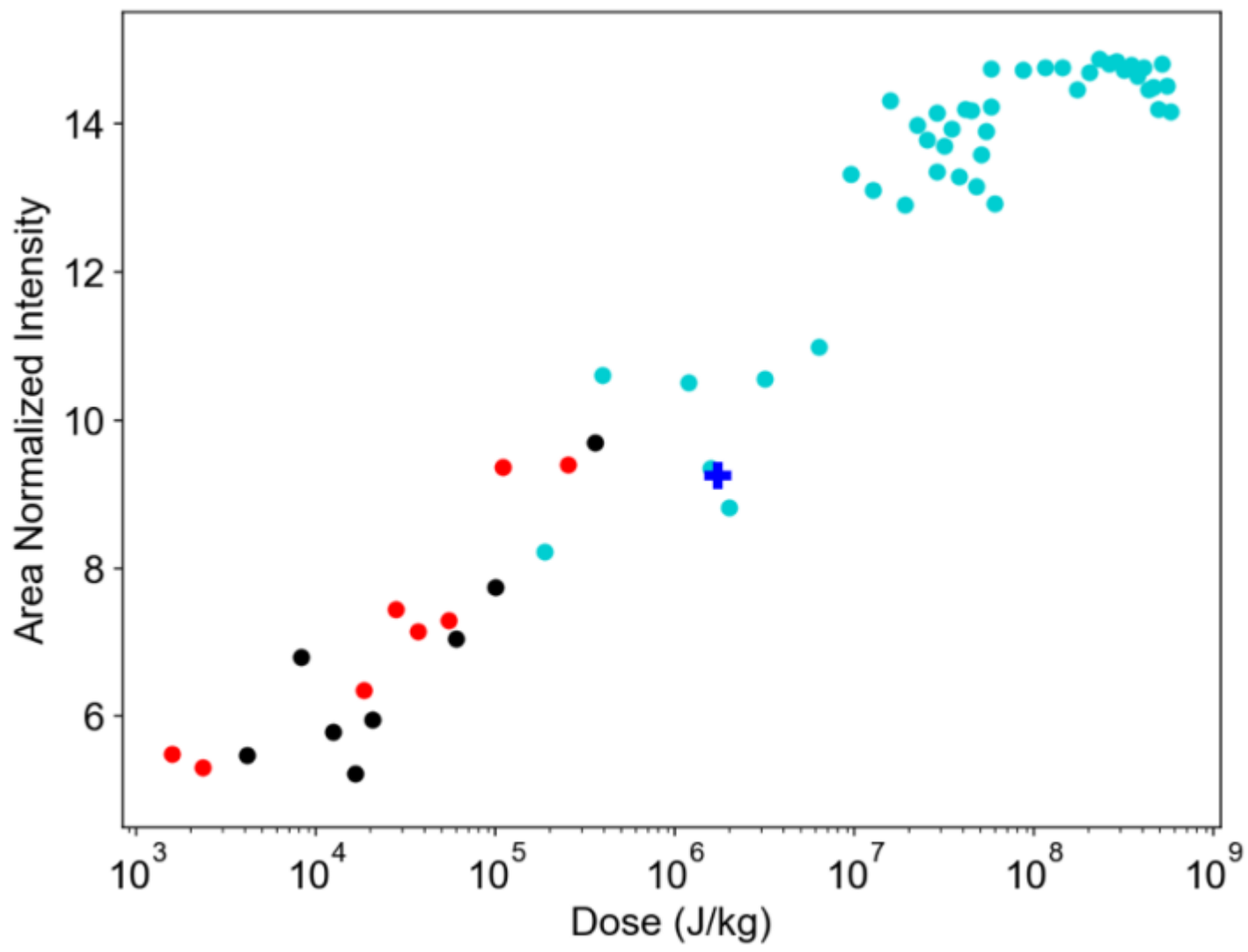

Figure S7. Reproduction of Figure 2 with the 291 K data from CHESS shown in red, the 77 K data from CHESS shown in black and the 30 K ESRF data shown in light blue. Data from Castillo 2017 is shown as a dark blue plus.

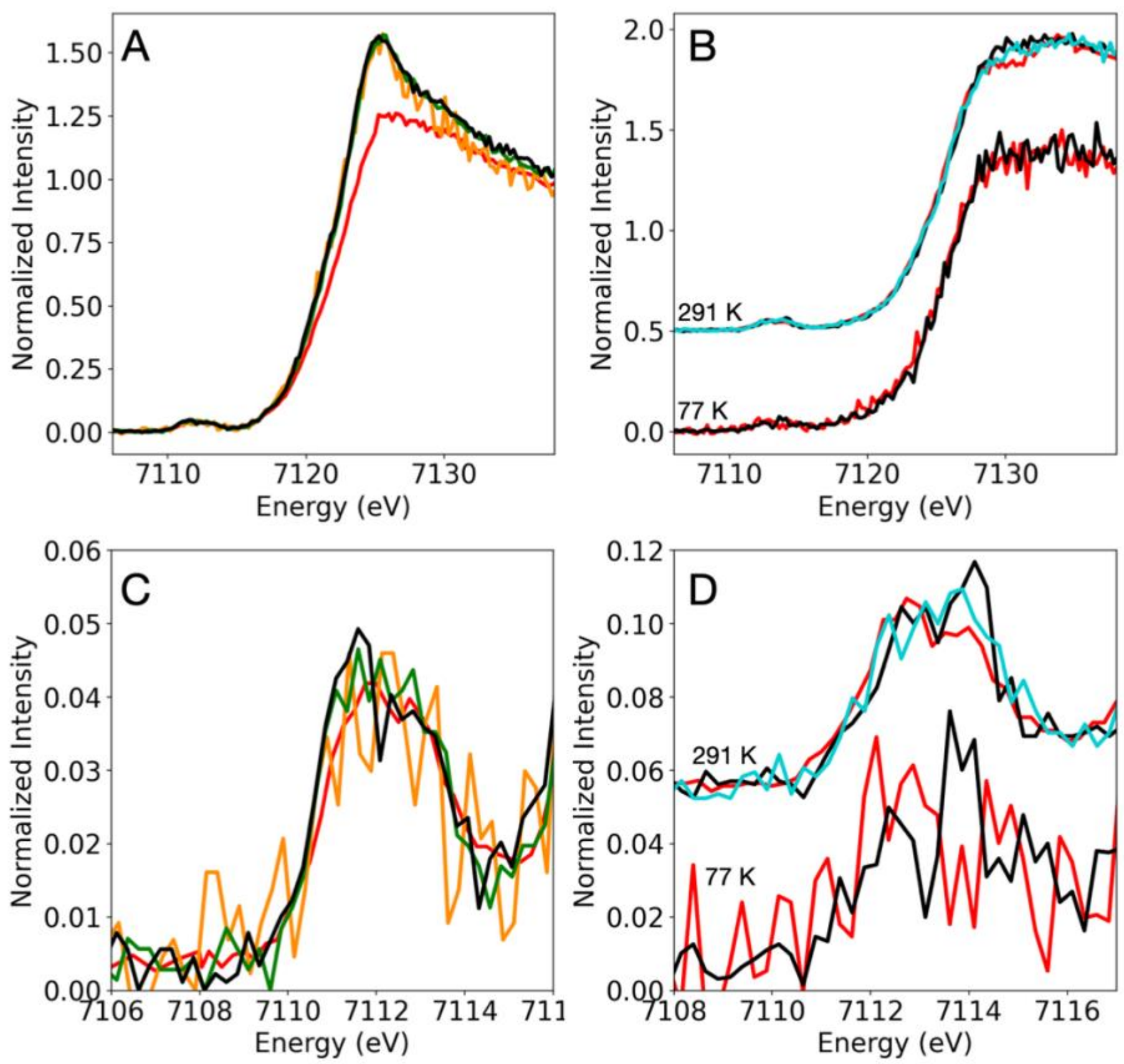

Figure S8. A. Unsmoothed HERFD XANES of MMOH-red alone at 77 K (in green) and 291 K (orange), and MMOH-red:MMOB at 77 K (black) and 291 K (red). B. Unsmoothed HERFD XANES of MMOH-ox (pH 7 in black) and MMOH-ox:MMOB (pH 7 in red, pH 7.5 in light blue) at 77 and 291 K. C and D are zoomed in on the pre-edge region of A and B, respectively.

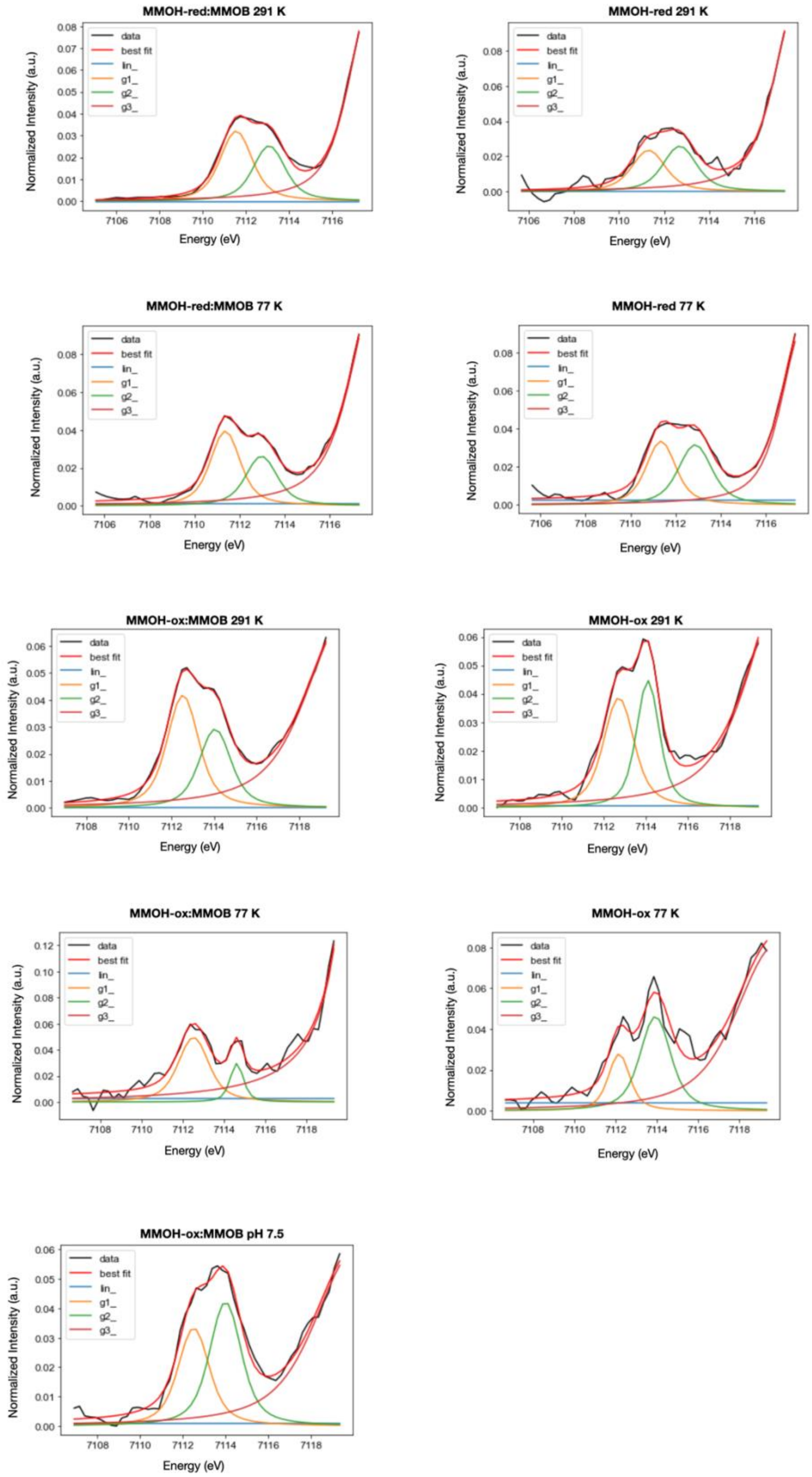

Figure S8. Pre-edge fits for all measured data. All data were fit using a linear baseline, and a pseudo-Voigt curve was used to account for the rising edge. The areas of the pre-edges were determined by the sum of the two pseudo-Voigt features used to fit the pre-edge features. For

MMOH-red data sets, the fitted range was 7105.6 to 7117.6 eV. For MMOH-ox data sets, the fitted range was 7106.9 to 7119.6 eV.

Computational Methodology

The MM models were constructed starting from two different crystal structures for the MMOH-ox:MMOB complex[1] and MMOH-ox only[2] (see **Fig. S8**). For the first model (**Model A**), we extracted one monomer from the high-resolution XFEL structure of MMOH-ox:MMOB from OB3b (6YD0.pdb, 1.95 Å).[1] For the second model (**Model B**), the X-ray crystal structure of MMOH-ox (6VK6.pdb, 1.52 Å)[2] was used. The monomeric units were protonated using the H++ webserver[3] under physiological conditions. Standard protonation states were assigned to the titratable residues (–1 for Asp, Glu and +1 for Arg, Lys) and the protonation states of the ligands coordinated to the diiron active site were manually assigned; His147 and His246 were protonated at the N_ε position. The active site ligands were renamed as shown in **Fig. S9** and the *antechamber* module[4] was used to assign the atom types to all the ligand atoms. All the crystal waters were retained.

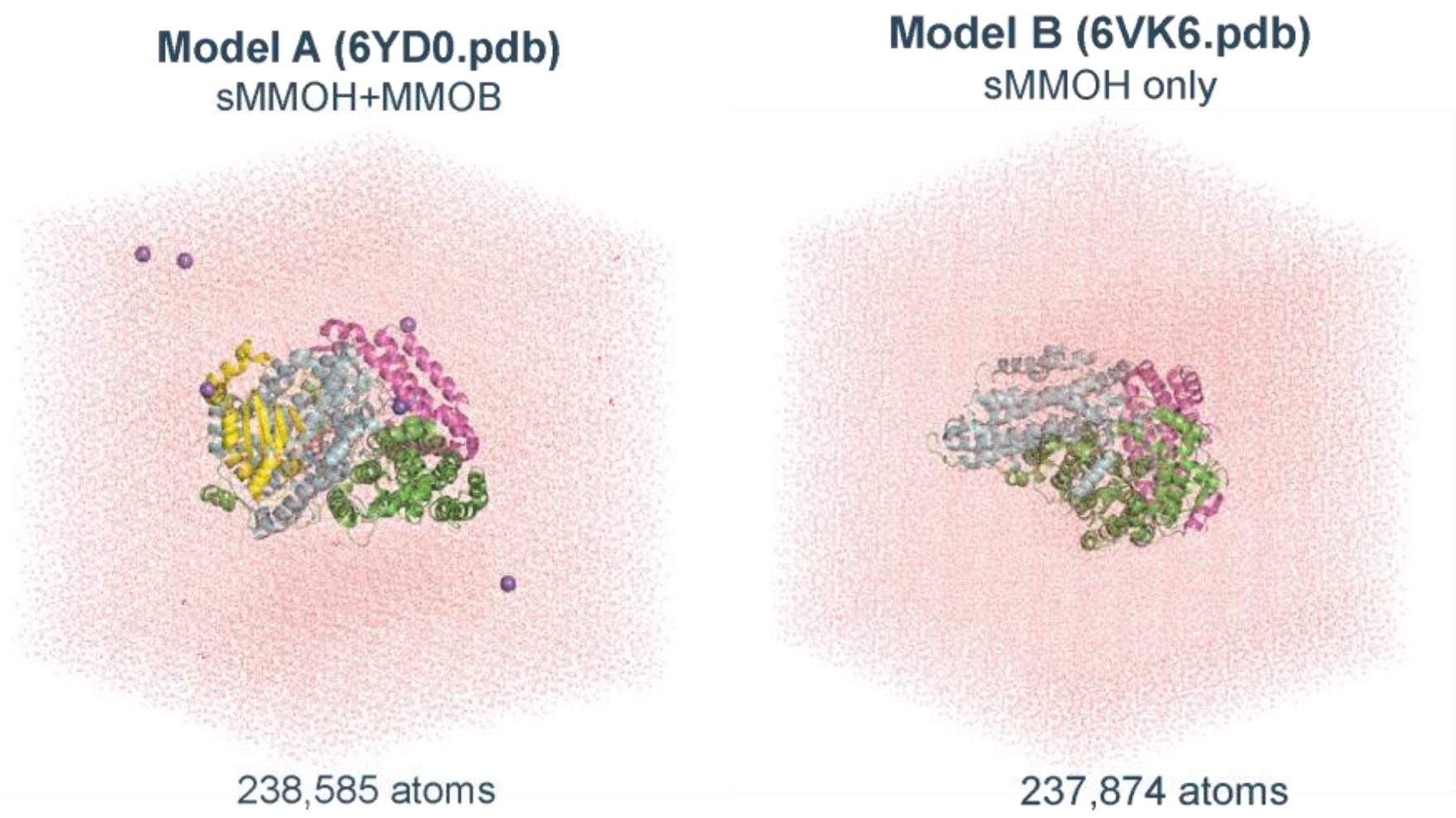

Fig S9. Depiction of the complete setup of **(a)** MMOH:MMOB **(b)** MMOH only (right), solvated within the TIP3P water box and counterions employed in classical MD simulations and QM/MM calculations throughout this work.

The complete protein system was solvated (in water) in a truncated octahedron box, with a minimum of 15 Å between the solute and box edge. Appropriate number of counterions were added randomly to maintain an overall neutral charge each setup. The final simulation setup consisted of 238,585 atoms for MMOH:MMOB and 237,874 atoms for MMOH only, as shown in **Fig. S8**.

Force Field Parameters

A bonded model[5] is used for deriving the force-field parameters of the diiron site based on a small QM cluster extracted from the MMOH:MMOB protein (see **Fig. S9**). The cluster consisted of the diiron ions, along with the sidechains of 6 ligand residues (His147, His246, Glu144, Glu114, Glu209 and Glu243). The axial ligand to Fe1 and the bridging oxygens are modelled as waters. The sidechains of the ligand residues were terminated at the C_α–C_β bond and capped with hydrogen atoms for the QM cluster calculations. The charge on the QM system was computed using the MK-RESP[6-8] fitting methodology. The total charge of the QM cluster

was –2 and the Fe (III) centers were considered in their high-spin (S=5/2,5/2) states. As a first step, the hydrogen atoms were optimized at B3LYP/def2-TZVP[9-11] level of theory, whereas the non-hydrogen atoms were kept fixed at their crystallographic positions. The RESP charge calculation was performed on the resulting optimized geometry, at B3LYP/def2-SVP level of theory using ORCA 6.0[12, 13] and Multiwfn[14] programs. The total charge of the backbone atoms was restrained on the hydrogen link atom for each amino acid residue. During the generation of the topology file (*.prmtop*), the RESP charges of the backbone atoms (C, O, C_α, H_α, N, H) were assigned according to the original AMBER force field.[7, 15, 16]

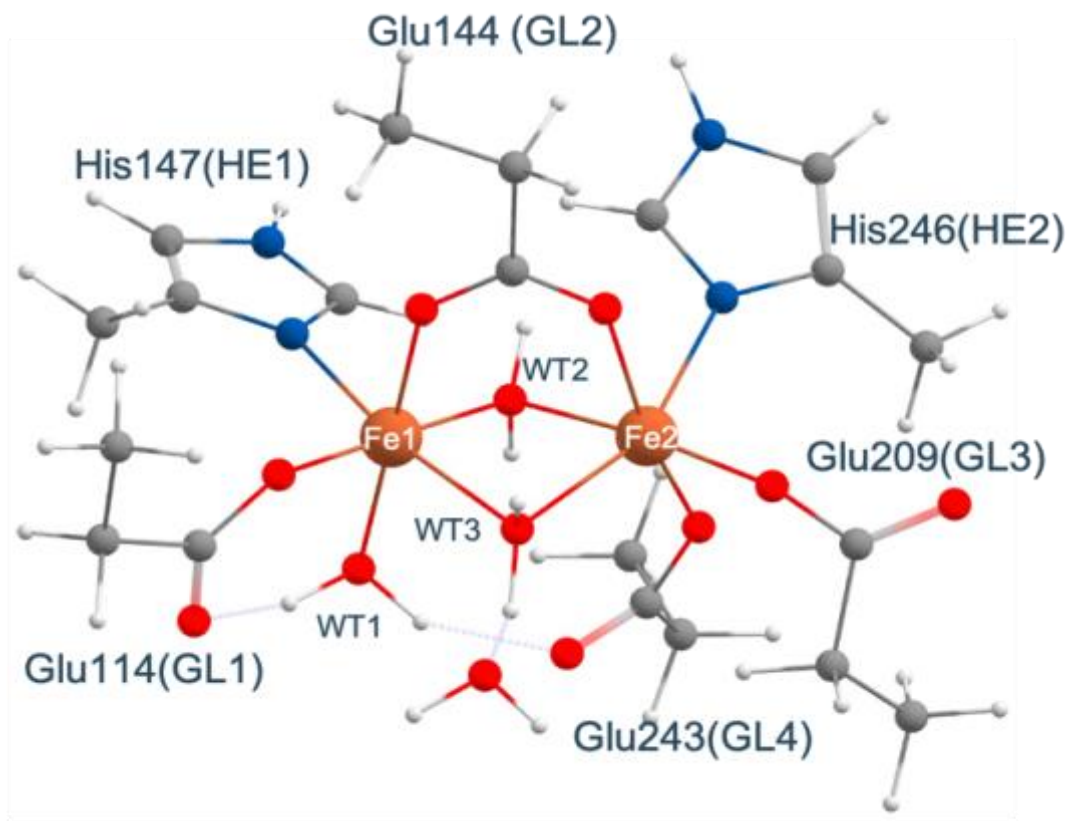

Fig. S10 QM cluster model used to derive the bonded parameters for the diiron site in sMMO (atomic coordinates derived from **6YD0.pdb**). The residue names used in the reconstructed MM model are labelled in parentheses. The residue number are labelled according to the original crystal structure.

The non-bonded parameters for Fe(III) were obtained from standard ion libraries in Amber compatible with the TIP3P water models.[17-19] The bonded parameters involving Fe(III) are arbitrarily defined to maintain the structural integrity of the diiron active site. The bonds between Fe and the coordinated ligand atoms were restrained with force constants of 300 kcal $mol^{-1}Å^{-2}$, whereas, the angles, with 100 kcal $mol^{-1}rad^{-2}$. The equilibrium bond lengths and angles were fixed according to the crystal structure (6YD0.pdb, 6VK6.pdb) geometry in each case. The remaining protein residues are defined using the standard Amber14SB force field[20] while the TIP3P model[17] is used for the solvent (water) molecules.

Molecular Dynamics Simulations

Step 1: Energy Minimization In the first step, all the hydrogen atoms of the system are energetically minimized for 2000 steps, which involves 1000 steps of steepest descent followed by 1000 conjugate gradient steps. The positions of heavy atoms were restrained with a force constant of 100 kcal $mol^{-1}Å^{-2}$. In the second minimization phase, all the hydrogen atoms, the solvent (waters) and ions were minimized for 2000 steps, keeping the rest of the protein restrained with a force constant of 100 kcal $mol^{-1}Å^{-2}$.

Step 2: Heating Dynamics (NVT) In the equilibration procedure, the systems are first heated from 0 to 298 K over 200 ps in the NVT ensemble. The temperature is controlled using the *Langevin* thermostat[21] with a collision frequency of 1.0 ps^{-1}. All the heavy atoms were harmonically restrained with a force constant of 50 kcal $mol^{-1}Å^{-2}$.

Step 3: Equilibration Dynamics (NPT) The final equilibration dynamics is performed in the NPT ensemble at physiological conditions (pressure ~1 atm, temperature ~298 K). The pressure was maintained using *Berendsen* barostat[22, 23] with a relaxation time of 1 ps. The

system was propagated in NPT for 500 ps until the average density of the simulation box was stable.

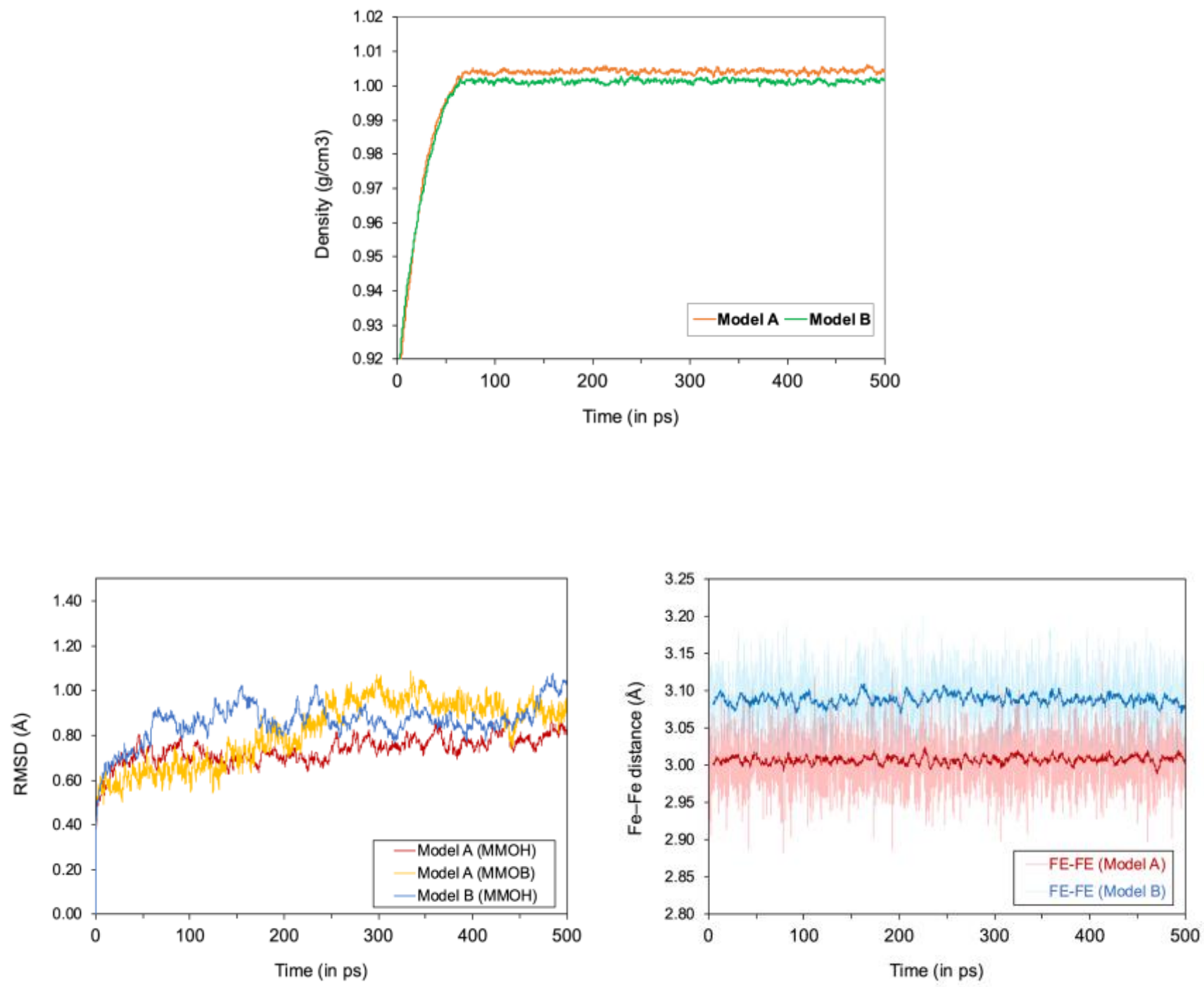

Fig. S11 (a) Variation of system density over the equilibration simulations for each setup. **(b)** RMSD of the C_α atoms of MMOH with (Model A) and without MMOB (Model B). **(c)** Variation of the average Fe–Fe distance over equilibration MD simulations. The original crystal structure distances are 3.04 Å for Model A and 3.13 Å for Model B, respectively

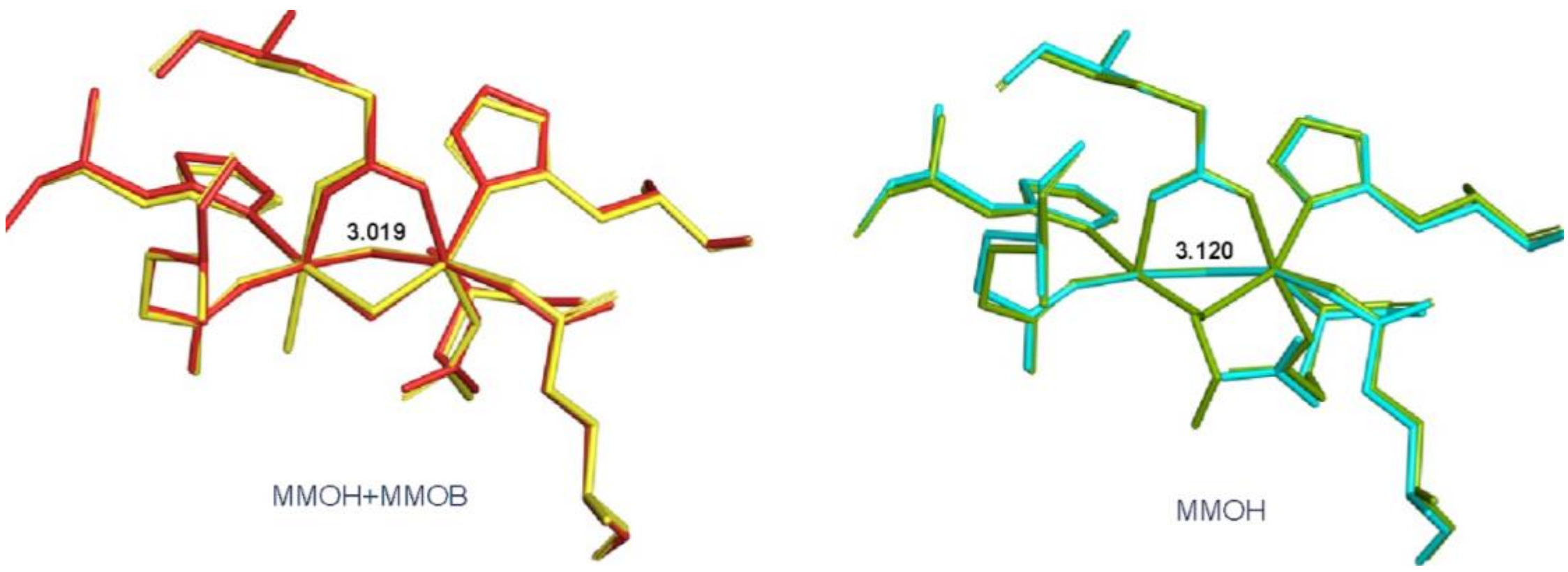

Fig. S12 Overlay of crystal structure and last frame of the equilibration simulations (to be used for QM/MM calculations). The MMOH:MMOB crystal structure is shown in yellow and MMOH in green. The active site from the last frame of MD simulation for Model A is represented in red and for Model B in cyan. The Fe–Fe distances indicated are from the last frame in each simulation.

A time-step of 2 fs was used throughout for integration of the equation of the motions. All bonds involving the hydrogen atoms were constrained using the SHAKE algorithm.[23] The Particle Mesh Ewald (PME) method[24, 25] is used to treat the long-range electrostatics with a non-bonded cut-off of 10 Å during the entire heating and simulation procedure. Throughout the equilibration procedure, all the heavy atoms within a 10 Å radius of the Fe–Fe center were harmonically restrained with a force constant of 50 kcal mol^{-1}Å^{-2}, to preserve the structural

integrity of the systems. The protein RMSD and Fe–Fe distance during the simulations was monitored using the Cpptraj module[26, 27] (**Fig. S10**). The last frame of the equilibration MD simulations for each setup is used for all QM/MM calculations in this work. The overall active site geometry and average Fe–Fe distance were conserved throughout the simulation process. All the MD and QM/MM setup, minimization and equilibration dynamics in this work are performed using the Amber20 program package. The results are compared to the original crystal structure in **Fig. S11**.

QM/MM Calculations

The active site in each case, is reconstructed based on varying protonation states and coordination numbers. All QM/MM calculations are performed using the electrostatic embedding scheme where the QM region is embedded within static point charges described by the MM force field. The QM/MM boundary is treated by the hydrogen link atom (LA) approach and charge shift is employed to avoid over-polarization of the QM atoms.[28] For the geometry optimizations the entire system is divided into an active (QM) part and a static MM part. The final QM region consisted of **251–254** atoms which included the Fe–Fe core along with all ligand residues, solvent derived ligands and the backbone/sidechains of the second sphere residues that maybe H-bonded to a residue in the first coordination sphere (**Fig.S12**).

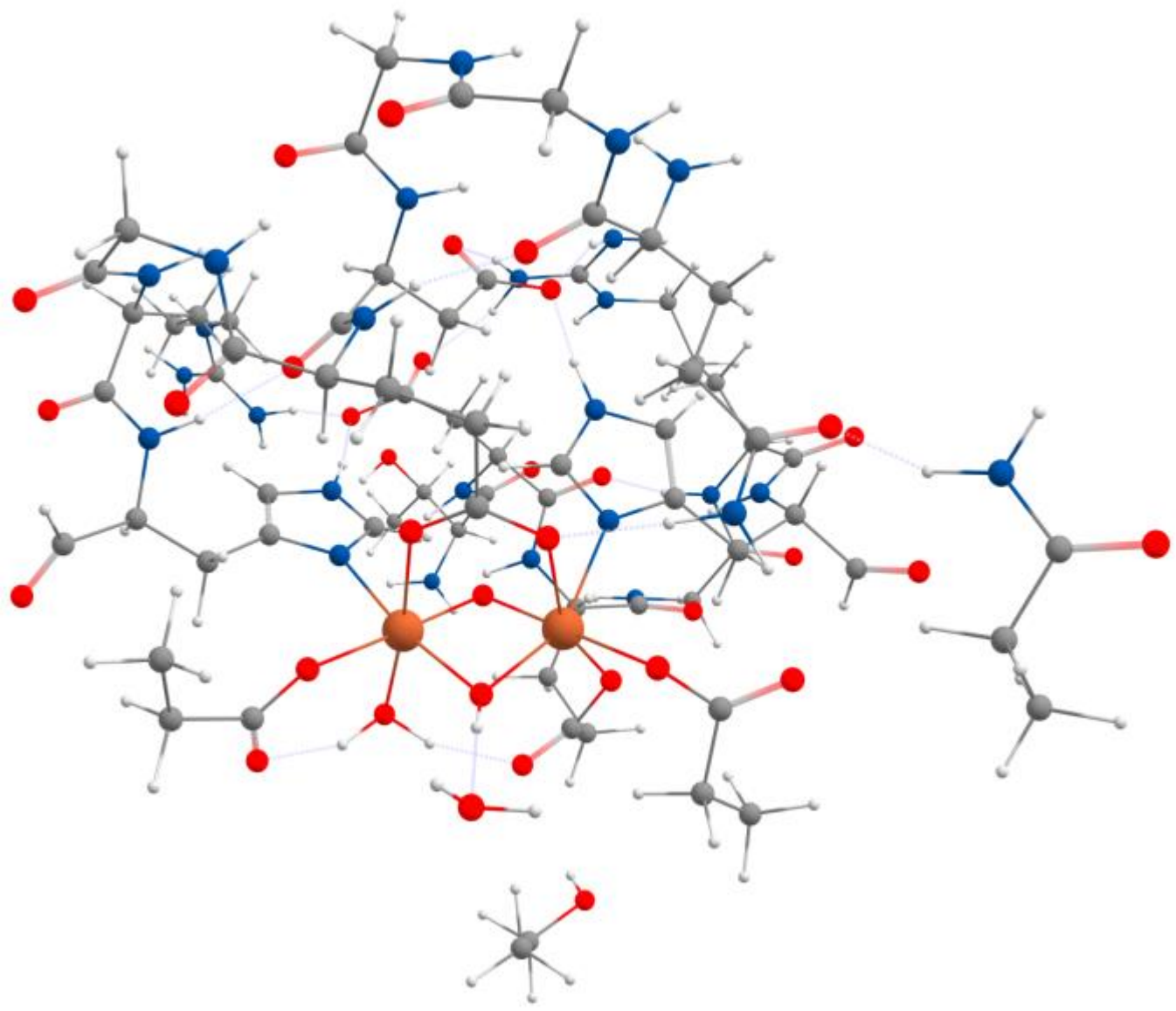

Fig. S13 The final QM region used in this work, indicating specific H-bonding interactions around the active site.

For the MMOH-ox models the Fe centers are treated as high spin with antiferromagnetic coupling (S = 5/2, 5/2) and the total charge of the QM system was -1 for ox-3 and 0 for ox-2. For MMOH-red, we optimized three alternative active site structures, for each model (A and B). The Glu243 carboxylate shift was introduced based on its crystallographic coordinates (6YDI and 6VK7). Additionally, we also reconstructed the active site based on the crystal structure of MMOH-red:MMOB (with 2 waters bound to Fe1 instead of just one).

Ox-3 (μ-O/OH) Model 2

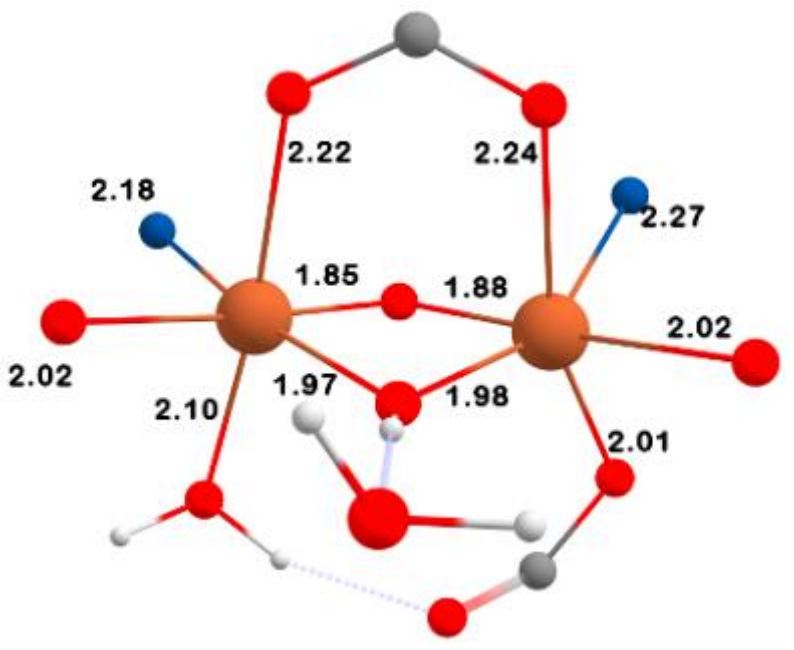

MMOH:MMOB

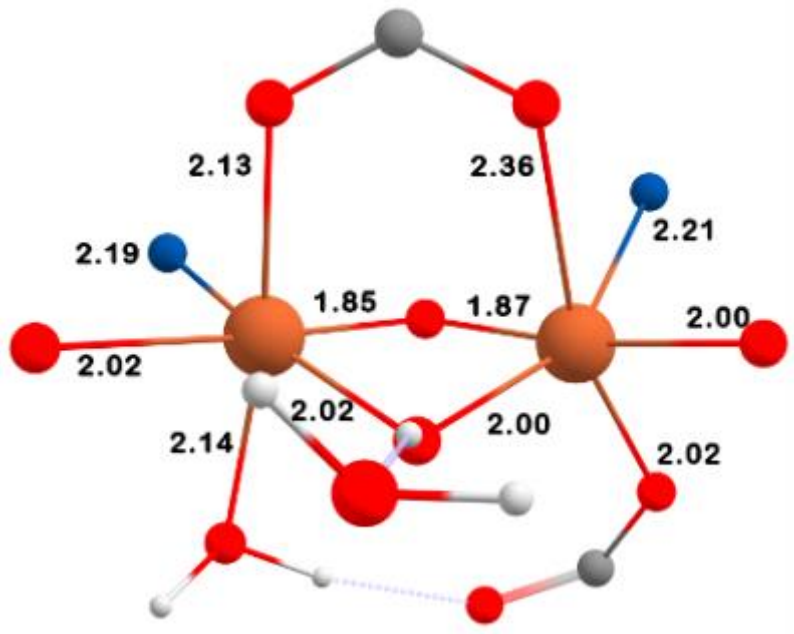

MMOH only

Ox-2 (μ-OH/OH) Model 1

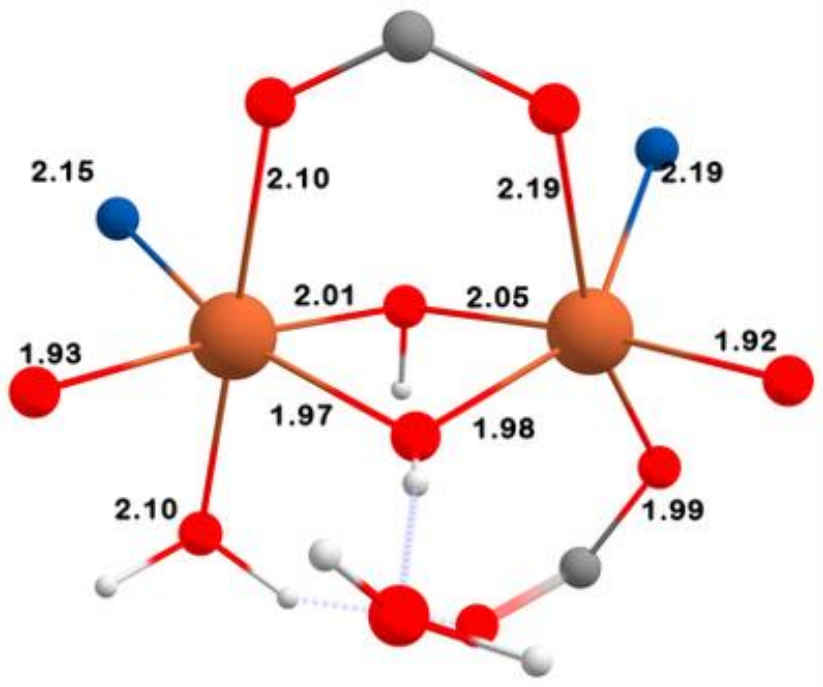

MMOH:MMOB

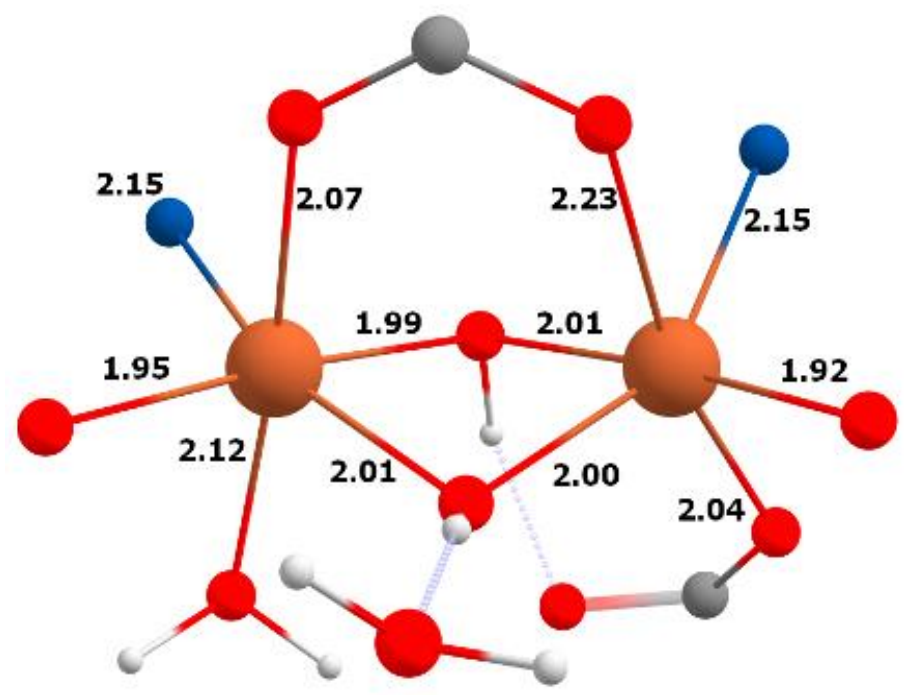

MMOH only

Figure S14. Bond lengths of sMMO-ox models.

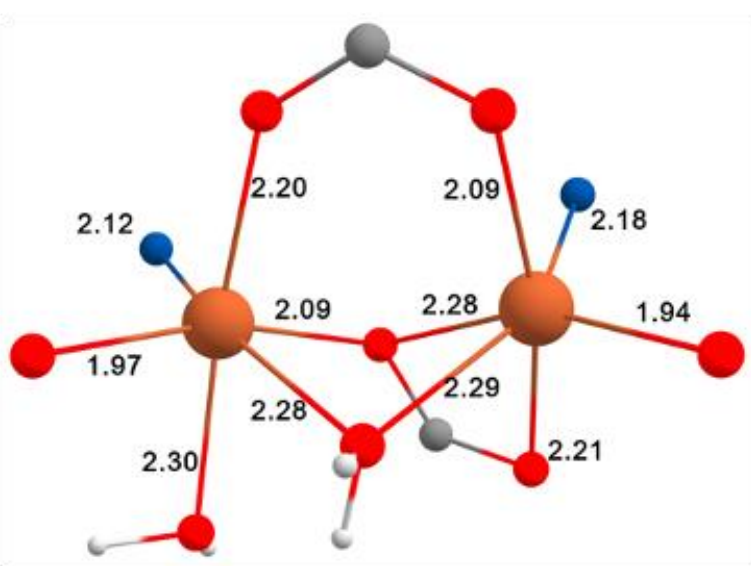

6,6

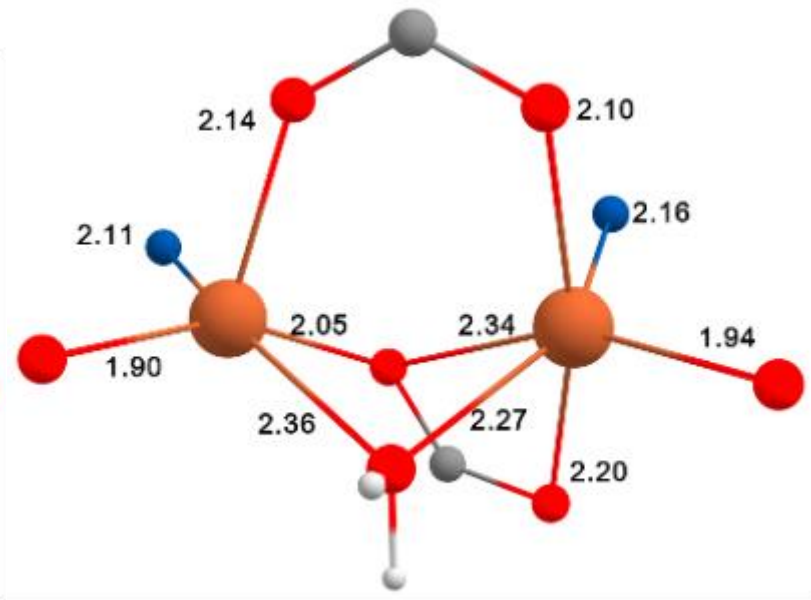

5,6

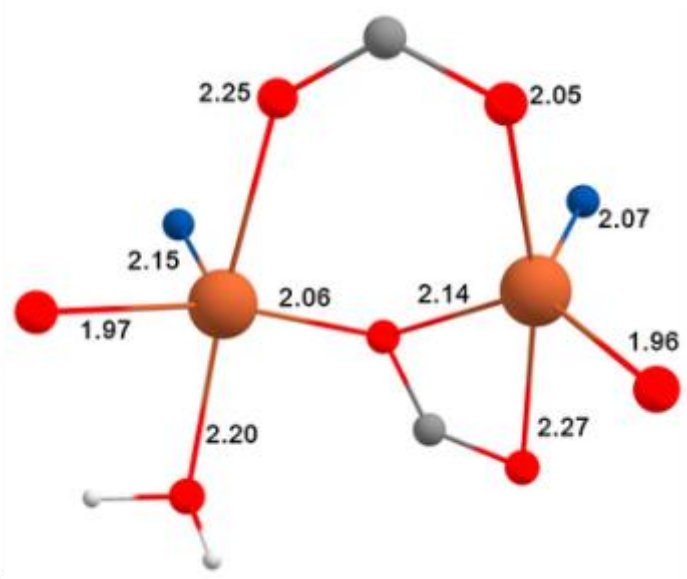

5,5

Figure S15. Bond lengths of MMOH-red:MMOB models.

Table S2. Mulliken spin populations for the two Fe centers in each optimized oxidized and reduced QM/MM model, calculated at the B3LYP/def2-TZVP level of theory.

Model	Fe1	Fe2
Ox1B	4.31	4.27
Ox1	4.31	4.27
Ox2B	4.24	4.23
Ox2	4.24	4.22
Red1B	3.80	3.77
Red2B	3.79	3.77
Red3B	3.80	3.77
Red1	3.81	3.77
Red2	3.81	3.78
Red3	3.79	3.77

Computational References

(1) Srinivas, V.; Banerjee, R.; Lebrette, H.; Jones, J. C.; Aurelius, O.; Kim, I.-S.; Pham, C. C.; Gul, S.; Sutherlin, K. D.; Bhowmick, A.; et al. High-Resolution XFEL Structure of the Soluble Methane Monooxygenase Hydroxylase Complex with its Regulatory Component at Ambient Temperature in Two Oxidation States. *J. Am. Chem. Soc.* **2020**, *142*, 14249-14266.
(2) Jones, J. C.; Banerjee, R.; Shi, K.; Aihara, H.; Lipscomb, J. D. Structural Studies of the Methylosinus trichosporium OB3b Soluble Methane Monooxygenase Hydroxylase and Regulatory Component Complex Reveal a Transient Substrate Tunnel. *Biochemistry* **2020**, *59*, 2946-2961.
(3) Gordon, J. C.; Myers, J. B.; Folta, T.; Shoja, V.; Heath, L. S.; Onufriev, A. H++: a server for estimating p Ka s and adding missing hydrogens to macromolecules. *Nucleic Acids Res.* **2005**, *33*, W368-W371.
(4) Case, D. A.; Aktulga, H. M.; Belfon, K.; Cerutti, D. S.; Cisneros, G. A.; Cruzeiro, V. W. D.; Forouzesh, N.; Giese, T. J.; Götz, A. W.; Gohlke, H.; et al. AmberTools. *J. Chem. Inf. Model.* **2023**, *63*, 6183-6191.
(5) Li, P.; Merz, K. M. Metal Ion Modeling Using Classical Mechanics. *Chem. Rev.* **2017**, *117*, 1564-1686.
(6) Li, P.; Song, L. F.; Merz, K. M., Jr. Parameterization of Highly Charged Metal Ions Using the 12-6-4 LJ-Type Nonbonded Model in Explicit Water. *J. Phys. Chem. B* **2015**, *119*, 883-895.
(7) Cornell, W. D.; Cieplak, P.; Bayly, C. I.; Gould, I. R.; Merz, K. M.; Ferguson, D. M.; Spellmeyer, D. C.; Fox, T.; Caldwell, J. W.; Kollman, P. A. A Second Generation Force Field for the Simulation of Proteins, Nucleic Acids, and Organic Molecules. *J. Am. Chem. Soc.* **1995**, *117*, 5179-5197.
(8) Bayly, C. I.; Cieplak, P.; Cornell, W.; Kollman, P. A. A well-behaved electrostatic potential based method using charge restraints for deriving atomic charges: the RESP model. *J. Phys. Chem.* **1993**, *97*, 10269-10280.
(9) Becke, A. D. A new mixing of Hartree–Fock and local density-functional theories. *J. Chem. Phys.* **1993**, *98*, 1372-1377.
(10) Becke, A. D. Density-functional thermochemistry. III. The role of exact exchange. *J. Chem. Phys.* **1993**, *98*, 5648-5652.
(11) Weigend, F.; Ahlrichs, R. Balanced basis sets of split valence, triple zeta valence and quadruple zeta valence quality for H to Rn: Design and assessment of accuracy. *Phys. Chem. Chem. Phys.* **2005**, *7*, 3297-3305.
(12) Neese, F. The ORCA program system. *WIREs Comput. Mol. Sci.* **2011**, *2*, 73-78.
(13) Neese, F. Software Update: The <scp>ORCA</scp> Program System—Version 6.0. *WIREs Comput. Mol. Sci.* **2025**, *15*.

(14) Lu, T.; Chen, F. Multiwfn: A multifunctional wavefunction analyzer. *J. Comput. Chem.* **2012**, *33*, 580-592.
(15) He, X.; Man, V. H.; Yang, W.; Lee, T.-S.; Wang, J. A fast and high-quality charge model for the next generation general AMBER force field. *J. Chem. Phys.* **2020**, *153*, 114502.
(16) Wang, J.; Wolf, R. M.; Caldwell, J. W.; Kollman, P. A.; Case, D. A. Development and testing of a general amber force field. *J. Comput. Chem.* **2004**, *25*, 1157-1174.
(17) Jorgensen, W. L.; Chandrasekhar, J.; Madura, J. D.; Impey, R. W.; Klein, M. L. Comparison of simple potential functions for simulating liquid water. *J. Chem. Phys.* **1983**, *79*, 926-935.
(18) Joung, I. S.; Cheatham, T. E., III. Molecular Dynamics Simulations of the Dynamic and Energetic Properties of Alkali and Halide Ions Using Water-Model-Specific Ion Parameters. *J. Phys. Chem. B* **2009**, *113*, 13279-13290.
(19) Joung, I. S.; Cheatham, T. E., III. Determination of Alkali and Halide Monovalent Ion Parameters for Use in Explicitly Solvated Biomolecular Simulations. *J. Phys. Chem. B* **2008**, *112*, 9020-9041.
(20) Maier, J. A.; Martinez, C.; Kasavajhala, K.; Wickstrom, L.; Hauser, K. E.; Simmerling, C. ff14SB: Improving the Accuracy of Protein Side Chain and Backbone Parameters from ff99SB. *J. Chem. Theory Comput.* **2015**, *11*, 3696-3713.
(21) Loncharich, R. J.; Brooks, B. R.; Pastor, R. W. Langevin dynamics of peptides: The frictional dependence of isomerization rates of <i>N</i>-acetylalanyl-<i>N</i>′-methylamide. *Biopolymers* **1992**, *32*, 523-535.
(22) Berendsen, H. J. C.; Postma, J. P. M.; van Gunsteren, W. F.; DiNola, A.; Haak, J. R. Molecular dynamics with coupling to an external bath. *J. Chem. Phys.* **1984**, *81*, 3684-3690.
(23) Ryckaert, J.-P.; Ciccotti, G.; Berendsen, H. J. C. Numerical integration of the cartesian equations of motion of a system with constraints: molecular dynamics of n-alkanes. *J. Comput. Phys.* **1977**, *23*, 327-341.
(24) Essmann, U.; Perera, L.; Berkowitz, M. L.; Darden, T.; Lee, H.; Pedersen, L. G. A smooth particle mesh Ewald method. *J. Chem. Phys.* **1995**, *103*, 8577-8593.
(25) Salomon-Ferrer, R.; Götz, A. W.; Poole, D.; Le Grand, S.; Walker, R. C. Routine Microsecond Molecular Dynamics Simulations with AMBER on GPUs. 2. Explicit Solvent Particle Mesh Ewald. *J. Chem. Theory Comput.* **2013**, *9*, 3878-3888.
(26) Roe, D. R.; Cheatham Iii, T. E. Parallelization of CPPTRAJ enables large scale analysis of molecular dynamics trajectory data. *J. Comput. Chem.* **2018**, *39*, 2110-2117.
(27) Roe, D. R.; Cheatham, T. E., III. PTRAJ and CPPTRAJ: Software for Processing and Analysis of Molecular Dynamics Trajectory Data. *J. Chem. Theory Comput.* **2013**, *9*, 3084-3095.
(28) Senn, H. M.; Thiel, W. QM/MM Methods for Biomolecular Systems. *Angew. Chem. Int. Ed.* **2009**, *48*, 1198-1229.

Additional Data

Table S3. IWAE and areas of individual peaks from the pre-edge fits in Figure S8. The fit error in the IWAE and areas of the individual peaks is determined by the uncertainty in the position and intensity of the fit. The fit error in the total area is the combined error of the areas of peak 1 and peak 2. In order to best account for the impact of the signal to noise ratio of individual spectra on the fits and not propagate error due to cross-correlating factors in the fit, the error of the total IWAE is not the propagation of the error of individual peaks. Rather, the error of the total IWAE is determined by the combined error of the total fit (achieved by running a Monte Carlo simulation varying the starting position of the peaks) and the error due to the relative noise of each spectrum (found by running a Monte Carlo simulation with randomized noise based on the signal to noise ratio of each spectrum).

Spectrum	Area			IWAE (eV)		
	Peak 1	Peak 2	Total	Peak 1	Peak 2	Total
MMOH-red 291 K	5.2 ± 3.0	5.7 ± 3.2	10.9 ± 4.4	7111.31 ± 0.27	7112.70 ± 0.26	7112.01 ± 0.33
MMOH-red 77 K	6.6 ± 1.4	7.0 ± 1.5	13.6 ± 2.1	7111.35 ± 0.08	7112.87 ± 0.10	7112.09 ± 0.25
MMOH-red:MMOB 291 K	7.1 ± 1.0	5.6 ± 1.0	12.7 ± 1.4	7111.55 ± 0.06	7113.08 ± 0.08	7112.20 ± 0.09
MMOH-red:MMOB 77 K	7.8 ± 0.8	5.5 ± 1.0	13.3 ± 1.3	7111.37 ± 0.04	7112.97 ± 0.07	7112.00 ± 0.17
MMOHox 291 K	8.4 ± 1.3	7.4 ± 1.1	15.9 ± 1.7	7112.71 ± 0.07	7114.09 ± 0.04	7113.35 ± 0.10
MMOHox 77 K	4.4 ± 1.5	10.3 ± 2.1	14.7 ± 2.6	7112.17 ± 0.10	7113.93 ± 0.08	7113.38 ± 0.21
MMOHox:MMOB 291 K	9.3 ± 0.8	6.5 ± 0.8	15.8 ± 1.1	7112.54 ± 0.04	7114.06 ± 0.06	7113.18 ± 0.05
MMOHox:MMOB 77 K	11.0 ± 1.6	2.9 ± 0.9	14.0 ± 1.9	7112.52 ± 0.06	7114.62 ± 0.06	7112.97 ± 0.28
MMOHox:MMOB 291 K pH 7.5	7.3 ± 2.0	9.4 ± 2.1	16.7 ± 2.9	7112.51 ± 0.13	7113.99 ± 0.10	7113.33 ± 0.06

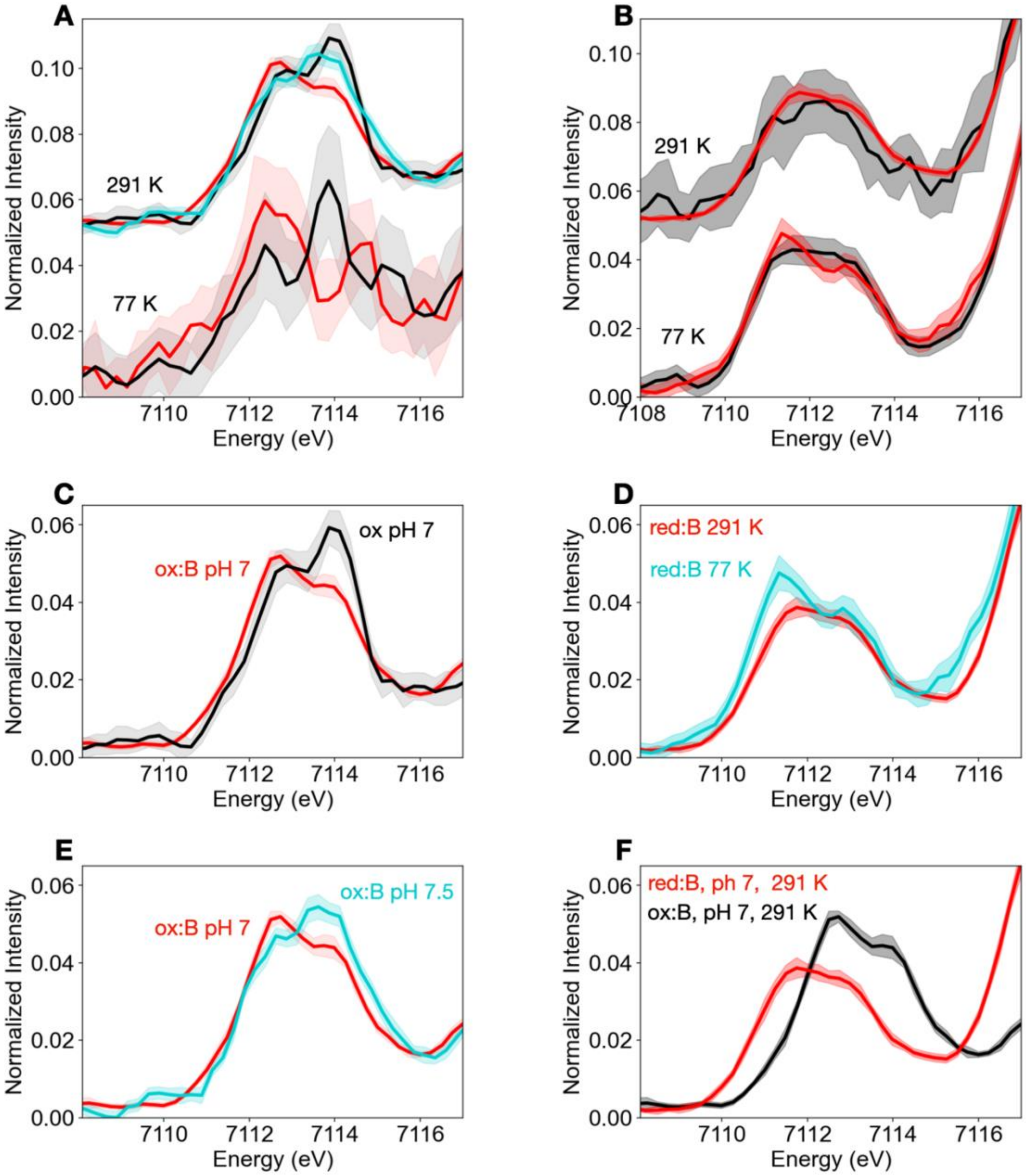

Figure S16. A series of graphs depicting various pre-edges and one standard error of the mean. Standard error of the mean is calculated by dividing the standard deviation by the square root of the number of spectra collected. A. Pre-edges of MMOH-ox alone (black) and MMOH-ox:MMOB at pH 7 (red) and pH 7.5 (teal) at 291 K (above) and 77 K (below) with one standard error of the mean shown as a shaded region. B. Pre-edges of the MMOH-red spectra at 291 K and 77 K (with MMOB shown in red, without MMOB in black) with one standard error of the mean shown as a shaded region. C. Pre-edge spectra and SEM of MMOH-ox at pH 7 with (red) and without (black) MMOB. D. Pre-edge spectra and SEM of MMOH-red:MMOB at 291 K (red) and 77 K (teal). E. Pre-edge spectra and SEM of MMOH-ox:MMOB at pH 7 (red) and pH 7.5 (teal). F. Pre-edge spectra and SEM of MMOH-ox:MMOB (black) and MMOH-red:MMOB (red) at 291 K, pH 7.

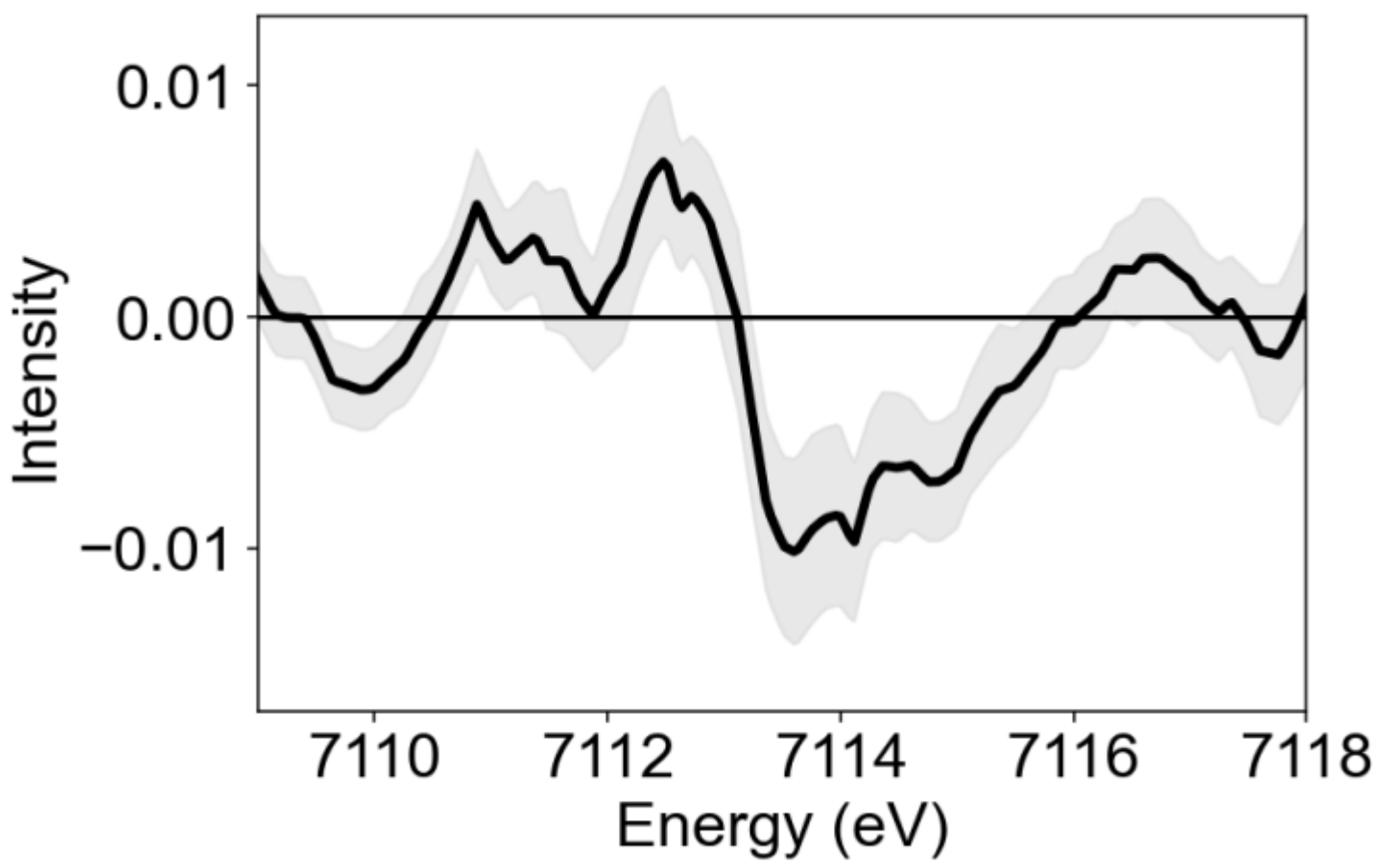

Figure S17. Difference spectrum of MMOH-ox:MMOB pre-edge with the pH 7.5 data subtracted from pH 7 shown in black, and the propagated standard error of the mean of the two data sets shown as the shaded gray region.

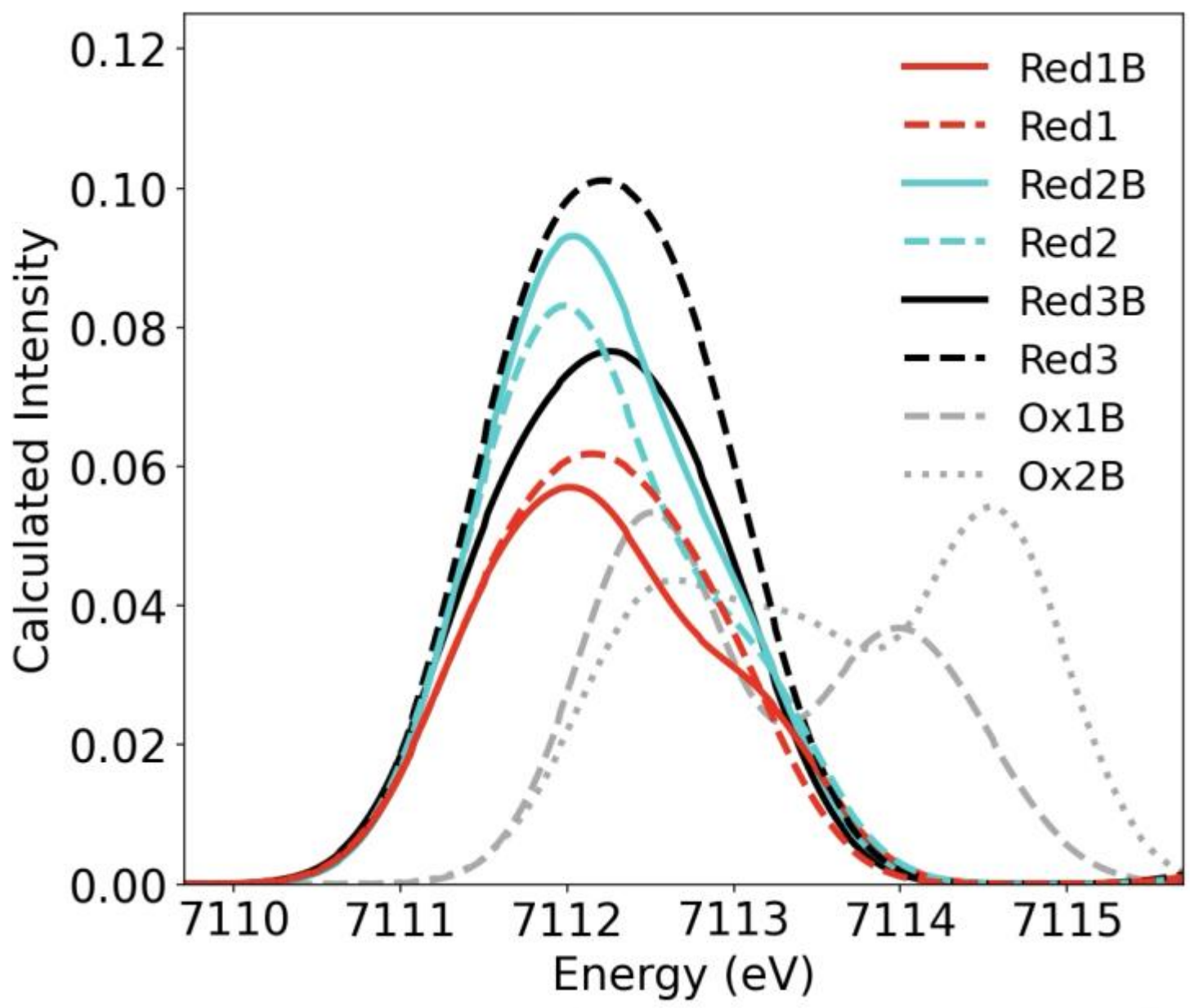

Figure S18. Comparison of all calculated MMOH-red and MMOH-red:MMOB models with MMOH-ox models Ox1B and Ox2B.

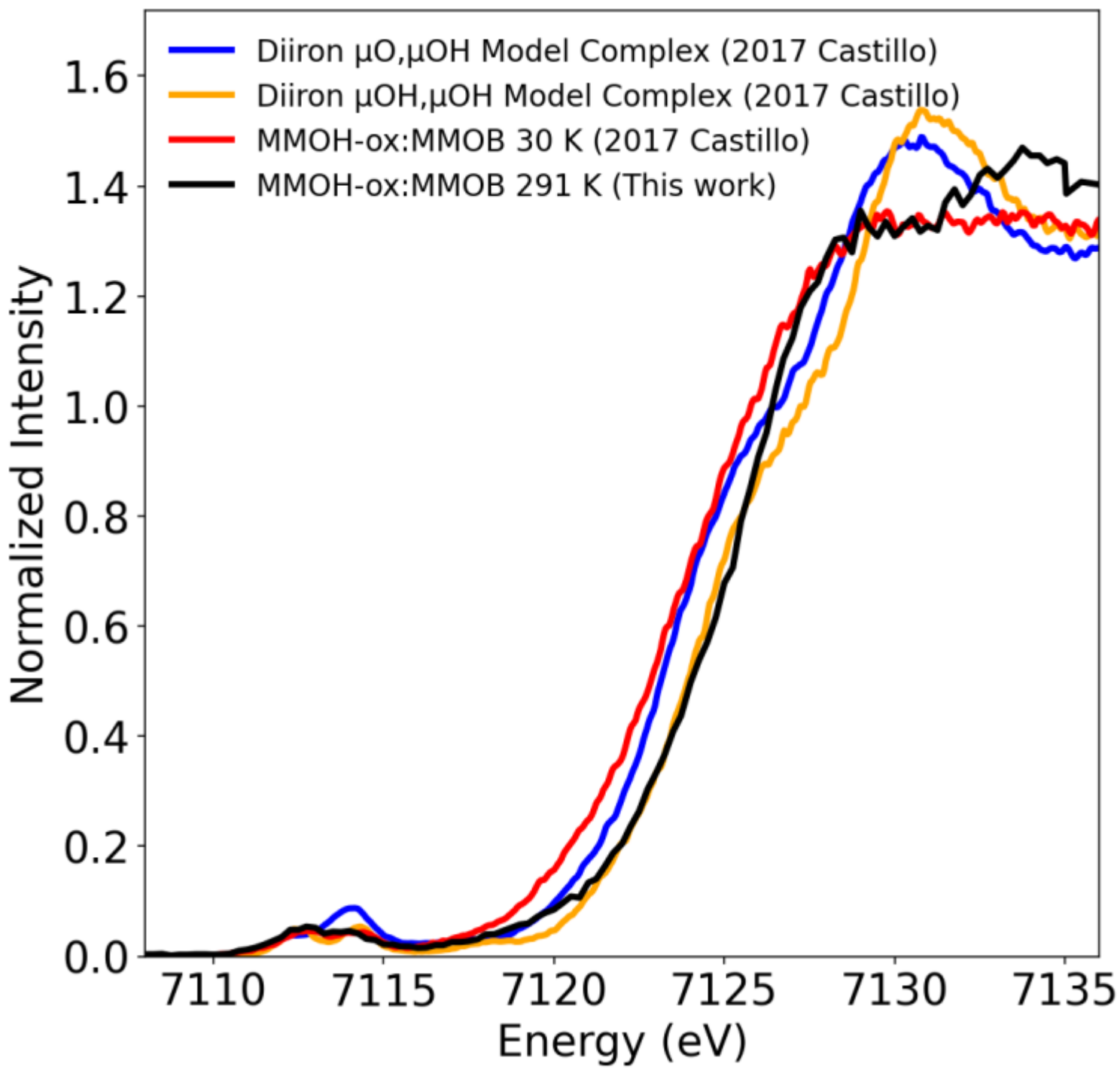

Figure S19. Comparison of HERFD XAS of diferric models from Castillo 2017[1], with previously published MMOH-ox:MMOB (now assigned semimet) and the current MMOH-ox:MMOB at 291 K. Select spectra eproduced from [1], copyright 2017 American Chemical Society.

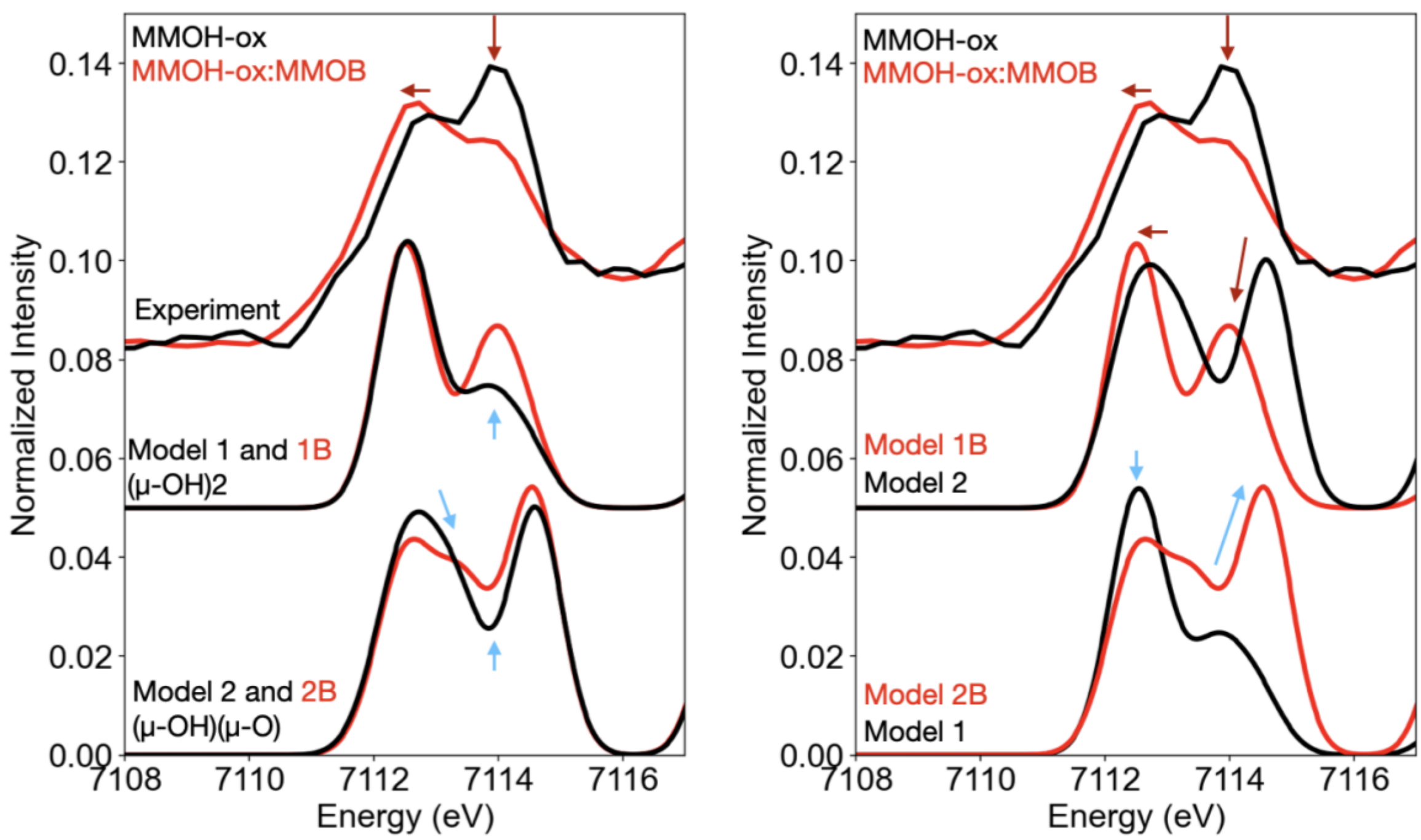

Figure S20. Comparison of experimental HERFD pre-edges of MMOH-ox (black) and MMOH-ox:MMOB (red) at pH 7 and all viable pairings of calculated pre-edges for MMOH-ox (black) and MMOH-ox:MMOB (red) models. Experimental changes upon MMOB binding are highlighted with dark red arrows. Calculated changes upon MMOB binding are highlighted by red arrows when they match experiment and blue arrows when changes upon MMOB binding do not match experiment.

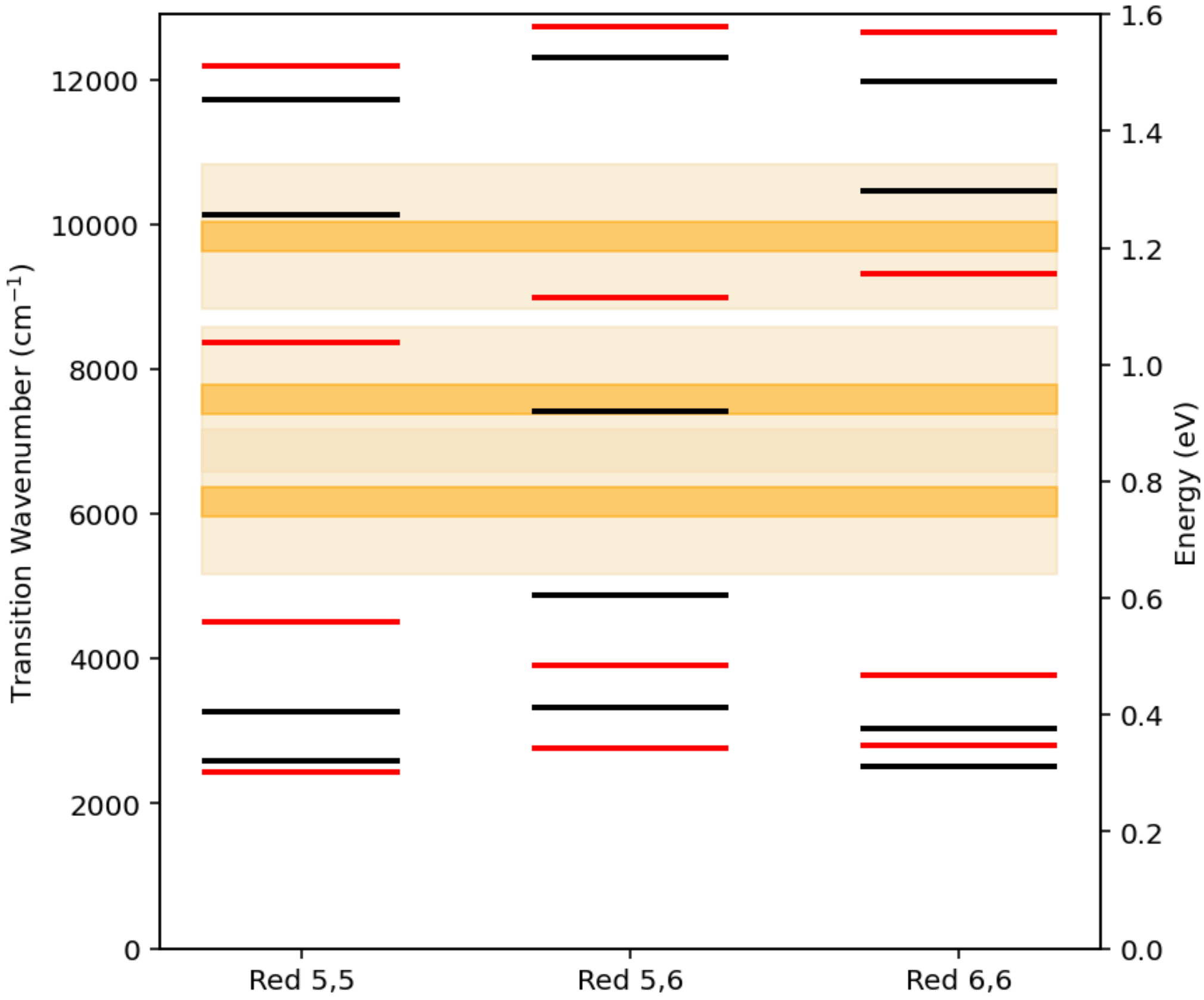

Figure S21. Energies of d-d transitions (shown in wavenumber and eV) from TDDFT absorption calculations of the MMOH-red:MMOB models. The d-d transitions from Fe1 are shown in black, and from Fe2 in red. The experimental MCD transitions as reported in Mitíc et. al. are shown with a ±200 cm^{-1} window (orange) and with the approximate full width half maximum of the features (beige).[2]

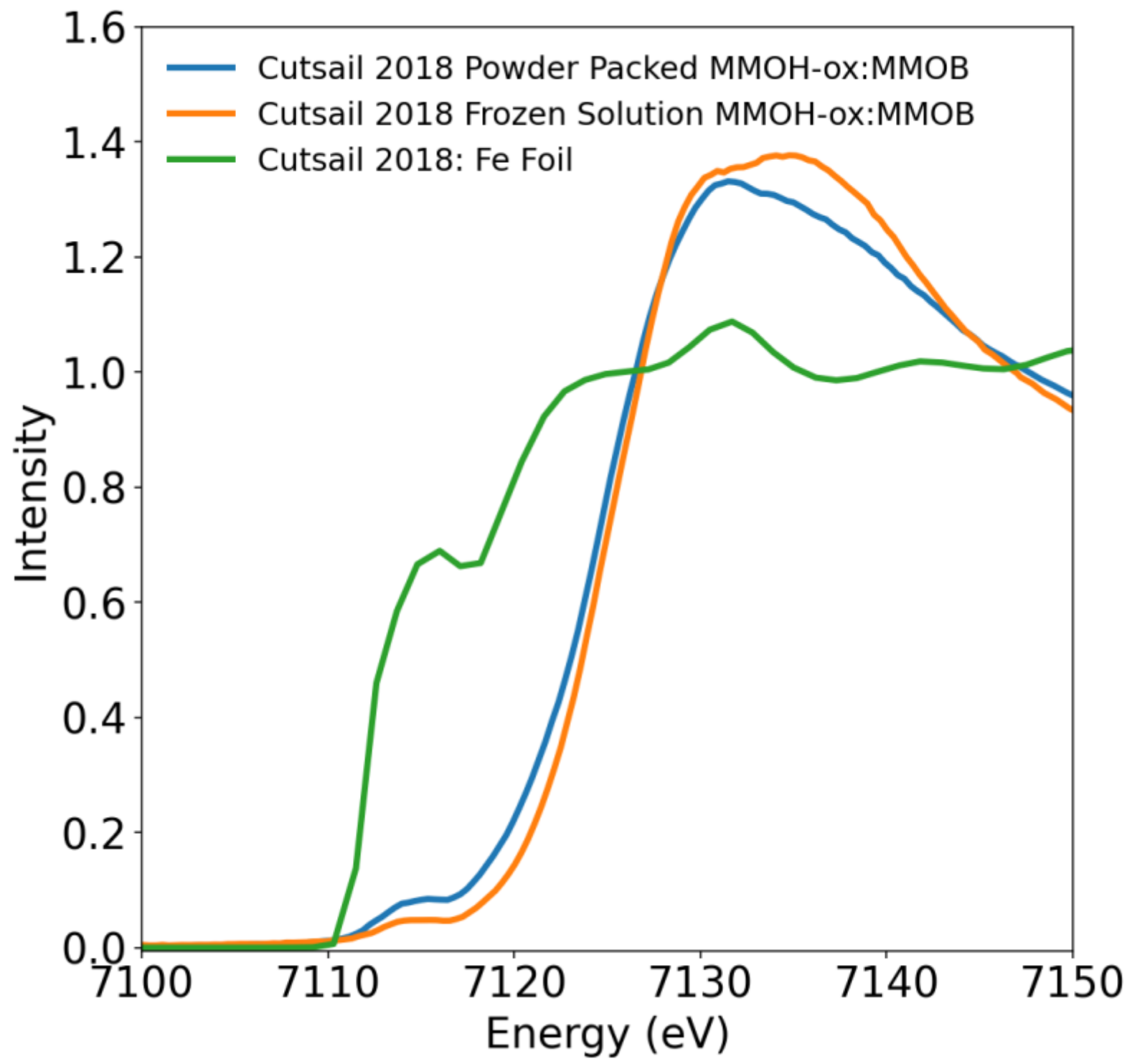

Figure S22. Reproduction of raw data from Cutsail 2018[3] Figure 8: partial fluorescence yield XAS of MMOH-ox:MMOB using two freezing methods, and the PFY XAS of an iron foil. Reproduced from [3], copyright 2018 American Chemical Society.

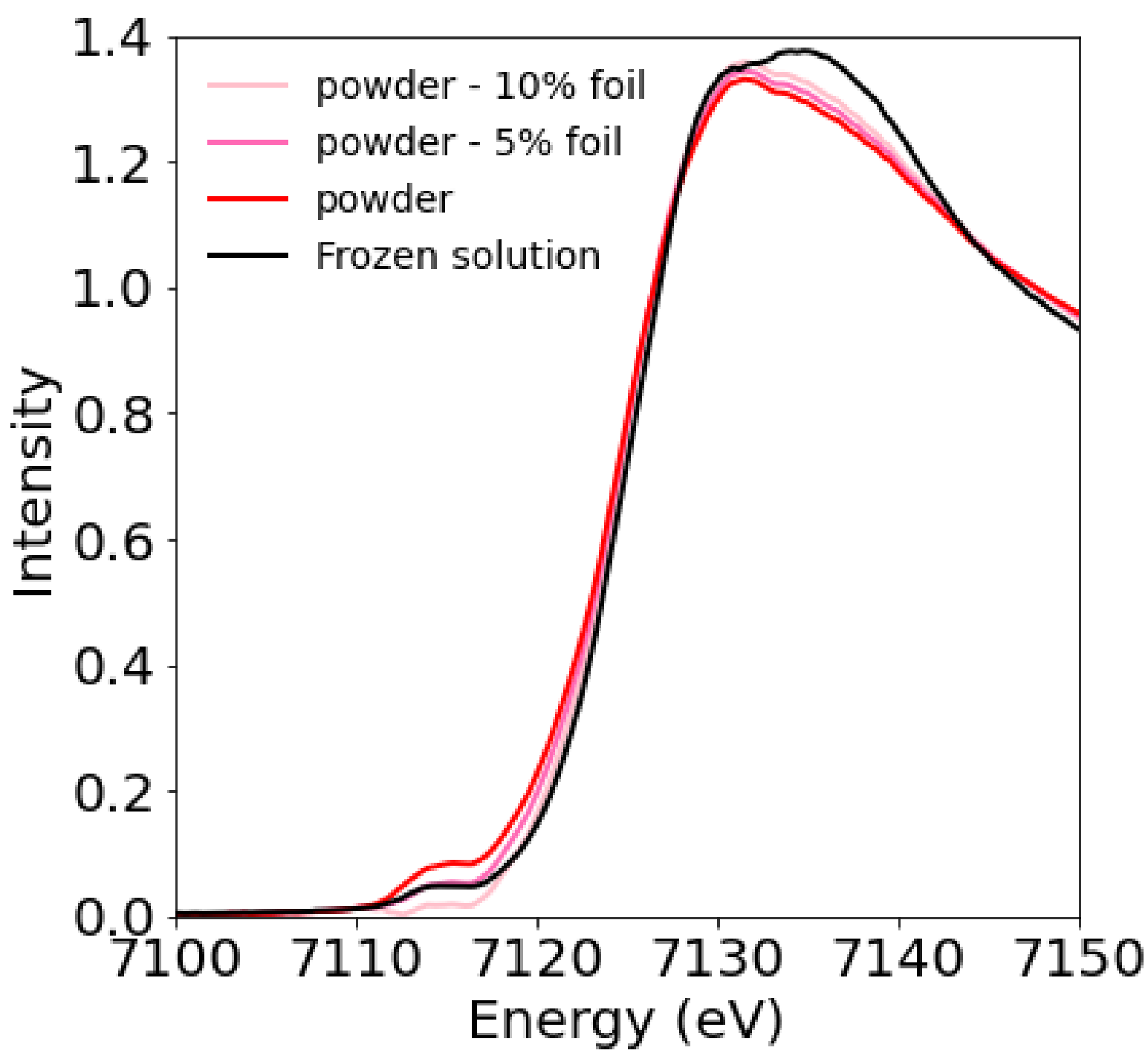

Figure S23. Comparison of Cutsail 2018[3] powder MMOH-ox:MMOB data contaminated with various amounts of iron foil and frozen solution MMOH-ox:MMOB data to highlight that the background contamination is not solely responsible for the edge shape change. Reproduced from [3], copyright 2018 American Chemical Society.

Additional Information References

(1) Castillo, R. G.; Banerjee, R.; Allpress, C. J.; Rohde, G. T.; Bill, E.; Que, L.; Lipscomb, J. D.; DeBeer, S. High-Energy-Resolution Fluorescence-Detected X-Ray Absorption of the Q Intermediate of Soluble Methane Monooxygenase. *J Am Chem Soc* **2017**.

(2) Mitić, N.; Schwartz, J. K.; Brazeau, B. J.; Lipscomb, J. D.; Solomon, E. I. CD and MCD Studies of the Effects of Component B Variant Binding on the Biferrous Active Site of Methane Monooxygenase. *Biochemistry* **2008**, *47* (32), 8386–8397. https://doi.org/10.1021/bi800818w.

(3) Cutsail, G. E.; Banerjee, R.; Zhou, A.; Que, L. Jr.; Lipscomb, J. D.; DeBeer, S. High-Resolution Extended X-Ray Absorption Fine Structure Analysis Provides Evidence for a Longer Fe···Fe Distance in the Q Intermediate of Methane Monooxygenase. *J Am Chem Soc* **2018**, *140* (48), 16807–16820. https://doi.org/https://pubs.acs.org/doi/10.1021/jacs.8b10313.
